# Genetic analysis of biopsy-related droplets in patients undergoing PGT-A and its potential application

**DOI:** 10.3389/fendo.2026.1734617

**Published:** 2026-05-04

**Authors:** Zhiqing Huang, Hang Shi, Yuzhi Chen, Xiaonuan Cai, Yanying Lin, Yiqin Chen, Wenchang Lian, Li Song, Beihong Zheng, Yan Sun

**Affiliations:** 1 Center of Reproductive Medicine, Fujian Maternity and Child Health Hospital, Fuzhou, China; 2 School of Basic Medical Sciences, Fujian Medical University, Fuzhou, China

**Keywords:** biopsy-related droplets, copy number variation, embryo selection, next-generation sequencing, preimplantation genetic testing, re-biopsy

## Abstract

**Purpose:**

Preimplantation genetic testing for aneuploidy (PGT-A) is essential for selecting embryos free from chromosomal abnormalities before transfer. However, challenges such as detection failures, mosaicism, and the limited accuracy of trophectoderm biopsy in representing the full chromosomal status of the blastocyst persist. Therefore, additional genetic information is needed to complement traditional PGT methods. This study investigated whether biopsy-related droplets (BRDs) contain detectable genetic material and evaluated their characteristics and potential clinical applications.

**Methods:**

A total of 318 BRDs were collected from 105 patients undergoing PGT-A. These droplets, generated during the biopsy process, underwent genomic DNA amplification and next-generation sequencing to detect copy number variations (CNVs).

**Results:**

The nucleic acid content of BRDs showed no significant correlation with blastocyst morphological grade, but was associated with the developmental day and chromosomal ploidy detected by PGT-A. BRDs of non-euploid blastocysts had higher nucleic acid content than those from euploid ones. Among 91 BRDs with successful CNV detection, 80.22% showed chromosomal ploidy results consistent with PGT-A. The clinical pregnancy rate for BRD-identified euploid blastocysts was significantly higher (84.62%) compared to those with failed or undetectable BRD results (58.00%). Notably, 8 of the 9 non-euploid blastocysts identified by BRD testing did not result in pregnancy. Furthermore, for 5 blastocysts that underwent secondary biopsy after initial PGT-A failure, chromosomal ploidy results from BRD analysis and secondary PGT-A were 80% consistent.

**Conclusion:**

BRD-based genetic analysis may serve as an auxiliary tool to PGT, improving embryo selection and transfer decisions by providing additional chromosomal insights.

## Introduction

Preimplantation genetic testing (PGT) is an assisted reproductive technology in which the chromosomal or genetic status of an embryo is analyzed based on a few cells obtained through biopsy before it is transferred into the uterus. This technology significantly reduces the likelihood of transferring chromosomally abnormal embryos or embryos that harbor a faulty gene, thereby enabling the birth of healthy offspring (1–3). PGT methods can be divided into three main categories: PGT for aneuploidy (PGT-A), PGT for structural rearrangements (PGT-SR), and PGT for monogenic disorders (PGT-M). PGT-A involves screening for embryonic chromosomal aneuploidy (1–3), serving as the basis of the PGT method. It is primarily recommended for women of advanced maternal age (≥38 years), those with unexplained recurrent spontaneous abortion or recurrent implantation failure, and couples in which the male partner has severe teratospermia, in order to select embryos without significant chromosomal abnormalities for transfer, thereby improving pregnancy outcomes and reducing the risk of miscarriage.

However, even without considering the invasive nature of biopsy and the fact that some embryos may undergo self-repair or mutation during development, PGT still faces some other challenges. First, PGT yields a proportion (1%-2%) of test failures or suspected abnormal results (typically ~1–2% in our center and neighboring ART clinics, rarely exceeding 2%) (4–6), requiring discussion of whether another biopsy should be performed (5, 7). Second, it is unclear whether the test results of trophectoderm (TE) can accurately represent the chromosomal status of the inner cell mass (ICM) or the whole blastocyst (8–10). Third, the detection result of mosaic embryos (accounting for approximately 10–20% of all blastocysts) leads to a challenging decision of whether to proceed with the transfer (5, 7). These issues cannot be completely resolved with biopsy-based genetic testing alone; additional information is needed to support further analysis.

Trophectoderm biopsy is performed in a droplet on a specialized biopsy dish. Commonly used biopsy methods include the laser method, the mechanical method, or a combination of the two (11). After the blastocyst is transferred into a fresh droplet and biopsied for several to more than ten minutes, the biopsied blastocyst is transferred to a temporary storage droplet before freezing, and the biopsy cells are collected into sample tubes for genetic testing. Since biopsy is an invasive procedure, genetic samples (such as blastocoelic fluid) could be largely introduced into the related operational droplets. To date, there have been no published reports on the use of the droplets from the blastocyst biopsy process as a medium for genetic detection.

**Figure 1:**
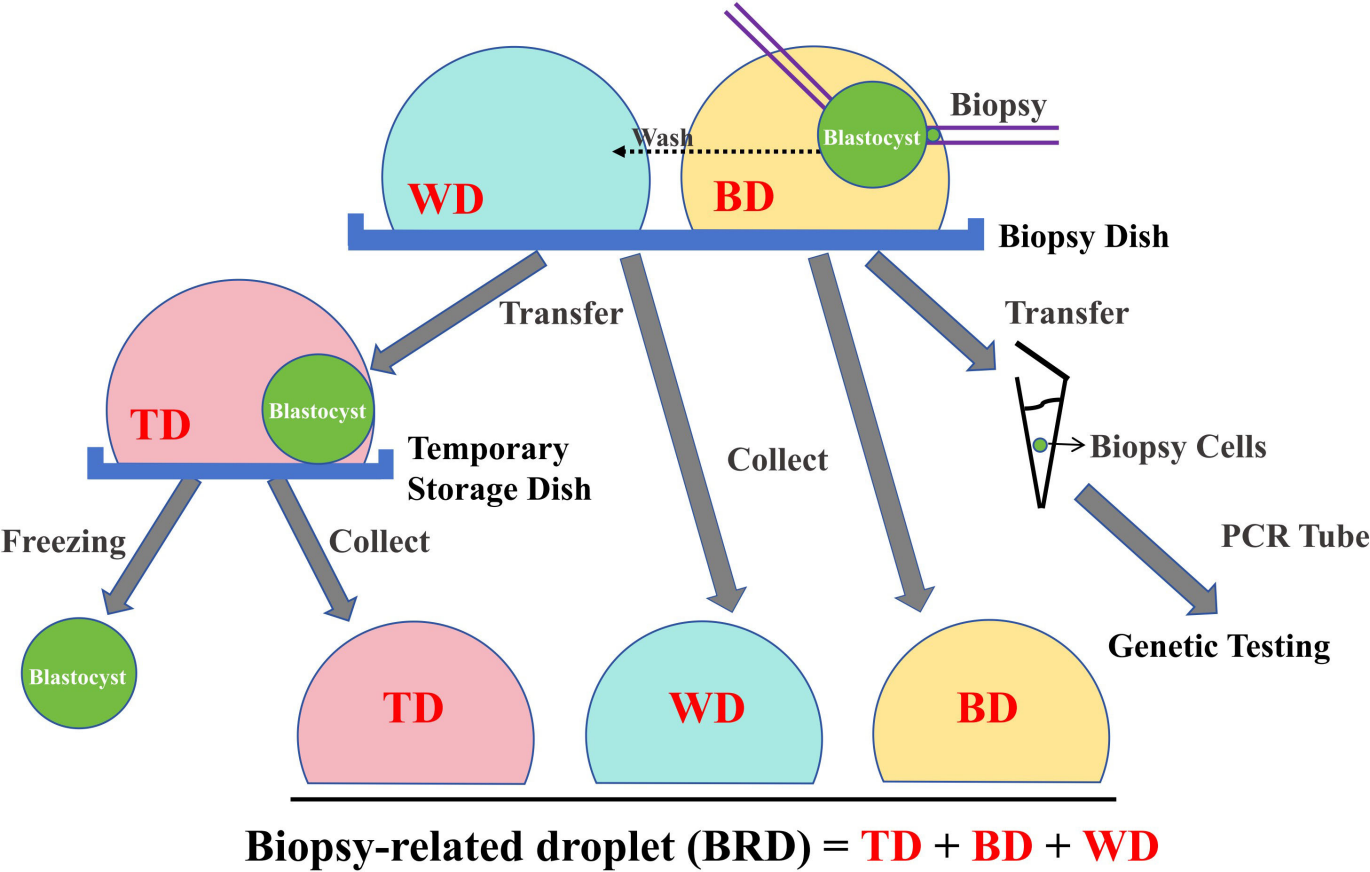
Schematic diagram of biopsy-related droplet (BRD) collection. During the blastocyst biopsy procedure, each blastocyst was sequentially exposed to three types of droplets: the biopsy droplet (BD), where the trophectoderm biopsy was performed; the washing droplet (WD), where the biopsied blastocyst and the biopsy cells were rinsed; and the temporary storage droplet (TD), where the biopsied blastocyst was placed before cryopreservation. After the procedure, the BD, WD, and TD corresponding to each blastocyst were fully aspirated and combined into a single sample tube, yielding 10–15 µl of biopsy-related droplet (BRD) for subsequent genomic analysis.

In this context, this study hypothesizes that biopsy-related droplets (BRDs), including the biopsy droplet, washing droplet, and temporary storage droplet generated during blastocyst biopsy, contain detectable genetic material from the blastocyst, which might offer additional insights into the chromosomal status of the embryo (**Figure 1**). The objective of the study is to assess whether BRDs can serve as a complementary source of genetic information to improve the accuracy of PGT, with potential clinical applications in embryo selection and transfer. In current clinical practice, TE results remain the primary basis for decision-making, whereas BRD analysis may provide supplementary information in selected cases.

## Methods

### Study participants

Patients who underwent PGT-A as part of assisted conception at the Reproductive Medicine Center of ** Hospital in 2024 were selected. A total of 105 couples who underwent PGT-A were randomly enrolled. Among them, 97.14% (102/105) of the female patients had secondary infertility. The ovulation induction regimens of 85.22% (98/115, 115 PGT cycles) and 13.04% (15/115) of the patients involved a gonadotropin-releasing hormone (GnRH)-A antagonist protocol and a GnRH-A ultra-long protocol, respectively.

The patients underwent standard protocols for ovulation induction, oocyte retrieval, semen optimization, *in vitro* fertilization, embryo culture and observation, blastocyst biopsy and genetic testing (1–3). After blastocyst biopsy, BRDs were collected for genetic testing. Additionally, patient’s information and embryological data were collected for subsequent correlation analyses.

### Experimental methods

#### *In vitro* fertilization, embryo culture and biopsy

After the oocytes were degranulated, metaphase II (MII) oocytes were collected and fertilized by intracytoplasmic sperm injection (ICSI) (micromanipulation system: Eppendorf, Hamburg, Germany; holding and injection pipettes: Cooper, San Ramon, U.S.). The sequential culture method was used for most *in vitro* embryo cultures, whereas 20 embryos from five cases were subject to the one-step culture method. In the sequential culture method, cleavage-stage embryos (Day 1–Day 3) were cultured in G1-PLUS medium (Vitrolife, Gothenburg, Sweden), and blastocyst-stage embryos (Day 4–Day 6) were cultured in G2-PLUS medium (Vitrolife, Gothenburg, Sweden), both in a benchtop incubator (COOK, Brisbane, Australia). In the one-step culture method, embryos were cultured in G-TL medium (Vitrolife, Gothenburg, Sweden) in a time-lapse incubator (GERI, Genea, Sydney, Australia). All embryos were cultured at 37 °C with 6% CO_2_ (V/V) and 5% O_2_ (V/V).

Blastocyst formation was observed on Day 5 (D5,125), Day 6 (D6,189) and Day 7 (D7, 4, totaling 193 for D6–7), approximately 116, 140 and 164 hours after insemination, respectively (**Table 1**). Blastocysts were evaluated using the Gardner grading system (12). Blastocysts with a score of 3BC, 3CB or higher were selected for biopsy. The biopsy dishes were prepared on the day of biopsy. Several biopsy droplets (BDs, 3–5 µL in volume) and washing droplets (WDs, 3–5 µL in volume) were made using GM-PLUS medium (Vitrolife, Gothenburg, Sweden) pre-equilibrated overnight. The dishes were covered with oil (Vitrolife, Gothenburg, Sweden) and incubated in a COOK incubator for at least 30 minutes before biopsy. Then, the day before biopsy, temporary storage droplets (TDs) with an approximate volume of 10 µL/droplet were prepared using G2-PLUS medium in temporary storage dishes, covered with oil, and placed in a COOK incubator to equilibrate overnight. Biopsies were performed using a combination of laser and mechanical methods. During blastocyst expansion, the laser was used to perforate the zona pellucida while avoiding the ICM. The biopsy needle was then used to aspirate and gently pull a small number of trophectoderm cells outward. During the pulling step, the laser was applied along the junction to cut the cells away, and 3–7 cells were eventually obtained. One BD was used exclusively for the biopsy of a single blastocyst, and the biopsy of each blastocyst generally took no more than 10 minutes.

**Table 1:** Analysis of factors influencing the CSLs of BRDs (N = 318).

Factors	Group values (mean ± SD or number)	t or F value	*P* value
Female Age (Years)	LCSL: 35.13 ± 4.52, HCSL: 35.04 ± 4.56	0.001	0.981
Male Age (Years)	LCSL: 36.20 ± 4.68, HCSL: 36.15 ± 4.87	0.201	0.654
Total GN Dose (IU)	LCSL: 2514.33 ± 640.36, HCSL: 2452.48 ± 584.45	0.007	0.936
GN Stimulation Duration (Days)	LCSL: 10.73 ± 1.42, HCSL: 10.67 ± 1.49	0.584	0.445
Number of Retrieved Oocytes	LCSL: 14.06 ± 7.93, HCSL: 14.17 ± 7.87	0.000	0.999
Number of MII Oocytes	LCSL: 11.38 ± 6.24, HCSL:11.36 ± 6.42	0.494	0.483
Number of High-Quality Day 3 Embryos	LCSL: 7.23 ± 4.56, HCSL: 7.43 ± 4.42	0.581	0.446
Number of Blastocysts Formed	LCSL: 5.90 ± 4.17, HCSL: 5.82 ± 4.02	0.541	0.463
Number of High-Quality Blastocysts	LCSL: 3.41 ± 2.46, HCSL: 3.19 ± 2.35	2.055	0.153
Duration of Female Infertility	<2 years: 187, ≥2 years: 131	2.077	0.150
Semen Quality	Normal: 257, Abnormal: 61	1.224	0.269
Blastocyst Developmental Day	Day 5: 125, Day 6 & 7: 193	5.580	0.018^*^
Blastocyst Morphological Grade	Good: 49, Fair: 164, Poor: 105	4.771	0.092
ICM Grade	A: 41, B: 265, C: 12	3.751	0.153
TE Grade	A: 15, B: 210, C: 93	1.489	0.475
Biopsy Operator	Operator 1: 142, 2: 71, 3: 77, 4: 28	2.929	0.403
CNV Result of Biopsy Cells	Euploid: 158, Non-euploid: 160	10.530	0.001^**^

BRD, Biopsy-related droplets; CNV, Copy number variation; CSL, Concentration of the sequencing library; GN, Gonadotropin; ICM, Inner cell mass; MII, Metaphase II; SD, Standard deviation; TE, Trophectoderm. “LCSL” indicates CSL < 0.5 ng/μL; “HCSL” indicates CSL ≥ 0.5 ng/μL. Total GN dose refers to the cumulative amount of gonadotropins administered during ovarian stimulation. GN stimulation duration refers to the number of days of gonadotropin administration. Number of high-quality Day 3 embryos refers to cleavage-stage embryos on Day 3 with a score of II/5 or above, based on the Istanbul Consensus. Number of high-quality blastocysts refers to blastocysts with a morphological grade of 4BB or higher, according to the Gardner grading system. Abnormal semen quality refers to the diagnosis of at least one of the following: oligozoospermia, asthenozoospermia, or teratozoospermia. Blastocyst developmental stage, blastocyst morphological grade, ICM grade, and TE grade were assessed according to the Gardner grading system. A good blastocyst morphological grade refers to embryos with both the ICM and TE graded B or above, with at least one being A; a fair grade refers to embryos with both ICM and TE graded B; a poor grade refers to embryos with either ICM or TE graded C. *P < 0.05; **P < 0.01.

### BRD collection, genome amplification and sequencing library construction

The sample collection and processing procedures are illustrated in **Figure 1**. As stated above, the blastocysts were biopsied in BDs. After biopsy, the biopsied blastocysts and biopsy cells were washed in the corresponding WDs in the biopsy dish. Then, the biopsied blastocysts were placed in the TDs before freezing. The biopsy cells were loaded into a sample preservation tube (PGT-XK-043, Yikon Genomics, Suzhou, China) for subsequent genetic testing. During the biopsy process, the corresponding blastocyst code of the BD and the TD were promptly labeled on the bottom of the operation dishes. After completion of the biopsy procedure, the oil in the biopsy dish and the temporary storage dish was removed using a pipette. For each biopsied blastocyst, the corresponding BD, WD and TD were aspirated completely, merging the three droplets into a single BRD of approximately 15 µL (**Figure 1**); for 7 embryos, only the BD and WD were obtained. The final BRD was collected into a sample preservation tube and labeled with the corresponding blastocyst code.

For the biopsy cells and their corresponding BRDs, the ChromInst Kit (Yikon Genomics, Suzhou, China) was used for genomic DNA amplification (Multiple Annealing and Looping-Based Amplification Cycles, MALBAC method) and NGS (Next-generation sequencing) library construction of copy number variation (CNV) according to the manufacturer’s instructions (11). The NGS libraries were purified using CMPure magnetic beads in the ChromInst Kit. The concentration of the purified library was determined using a Qubit 3.0 (Thermo Fisher, Waltham, USA). An equal volume of blank mixed droplet was used as the negative control, and commercially available trisomy 21 fibroblast cells (XK-007, Yikon Genomics, Suzhou, China) were used as the positive control.

### NGS and CNV analysis

Biopsy cells that yielded a purified NGS library concentration ≥ 5.0 ng/µL and BRDs that yielded a purified NGS library concentration ≥ 1.0 ng/µL were sent to JMDNA Biomedical Technology Co., Ltd (Shanghai, China) for sequencing with a DNBSEQ-T7 sequencer (BGI, Shenzhen, China) in PE150 paired-end mode and a sequencing volume of no less than 2 M reads (11). CNV analysis was performed using ChromGo software (Yikon Genomics, Suzhou, China) according to standard procedures (11). Additionally, the biopsy cells from 8 blastocysts (B19, B32, B40, B43, B48, B49, B117, B118, **Supplementary Table 1**) of 3 patients were sent to Berry Genomics Co., Ltd (Beijing, China) for CNV sequencing and analysis. Reports included chromosomal aneuploidy, fragment deletion/duplication of ≥ 4 Mb for TE and ≥ 10Mb for BRD, and mosaic abnormalities of 10 Mb and above with a proportion of ≥ 30%. Mosaicism was defined as a proportion of abnormal segments between 30% and 70%, euploidy was defined as a proportion of abnormal segments < 30%, and aneuploidy was defined as a proportion of abnormal segments > 70%. Non-euploidy includes mosaic or aneuploidy. The reference genomics used for CNV analysis was GRCh37/hg19.

### Statistical analysis

Data analysis was performed with SPSS 26.0 software. Quantitative data with a normal distribution and homogeneity of variance are presented as the means ± standard deviation ( $\bar{x}$ ± s), and intergroup comparisons were performed with the independent-samples t test. Categorical data are expressed as proportions or percentages (%), and intergroup comparisons were performed with the chi-square (X^2^) test or Fisher’s exact test. P < 0.05 was considered to indicate statistical significance, and P < 0.01 was considered to indicate high statistical significance.

## Results

### Concentration of the BRD-derived sequencing library

A total of 318 blastocysts from 115 oocyte retrieval cycles were included. Successful CNV detection was achieved for all TE samples; after the first biopsy for 313 blastocysts, and after the second biopsy for the remaining 5 blastocysts (5/318, 1.57%). The BRDs (BDs, WDs and TDs combined; **Figure 1**) of all the blastocysts were successfully acquired. The volumes of the BRDs were approximately 15 µL, and all the BRDs were used for genome amplification and NGS library construction of CNV. The average concentration of the purified NGS library (hereafter referred to as the “concentration of the sequencing library”, CSL) of the blank BRDs (i.e., negative controls) was 0.246 ng/µL (3 replicates). The CSL of 50.63% of the BRDs (161/318) was ≥ 0.5 ng/µL (approximately 2 times higher than the average CSL of the negative controls), while 51.55% of the BRDs (83/161) had a CSL ≥ 1.0 ng/µL. Using 0.5 ng/µL as the threshold, we classified the 161 blastocysts with CSL ≥ 0.5 ng/µL as the high-CSL group (HCSL) and the 157 blastocysts with CSL < 0.5 ng/µL as the low-CSL group (LCSL).

**Figure 2:**
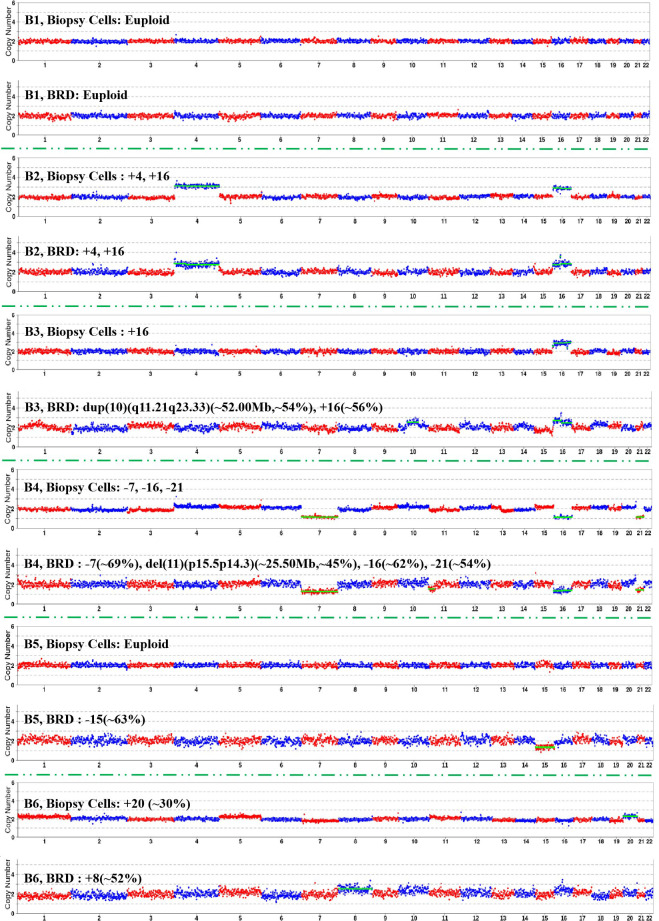
CNV profiles of biopsy cells and corresponding BRDs (6 representative samples).

83 purified NGS libraries with a CSL ≥ 1.0 ng/µL were subjected to NGS, among which the CNVs of 92.77% of the samples (77/83) were successfully analyzed (**Figure 2**, **Table 2** show the results of 6 representative samples; **Supplementary Table 1** shows the results of the remaining samples). In addition, among 20 BRD samples with a CSL < 1.0 ng/µL (Samples corresponding to blastocysts detected as euploid by PGT-A and had been transferred, including 12 samples with a CSL ≥ 0.5 ng/µL, and 8 samples with a CSL ≥ 0.35 ng/µL and < 0.5 ng/µL), CNVs were successfully detected in only 70% (14/20) (**Supplementary Table 1**). Of the 6 BRDs in which CNVs were not detected, 4 had CSLs below 0.5 ng/µL. CNVs were successfully detected in 83.33% (10/12) of BRDs with CSLs between 0.5 ng/µL and 1.0 ng/µL, and in 50% (4/8) of BRDs with CSLs between 0.35 ng/µL and 0.5 ng/µL. CNVs could be detected in the positive controls (+21, i.e., trisomy 21, in line with the theoretical result), but could not be detected in negative controls.

**Table 2:** CNV result consistency between biopsy cells and corresponding BRDs (6 representative samples).

Blastocyst code	CNV of biopsy cell	CSL (ng/µL)	CNV of BRD	Result consistency
B1	Euploid	15.16	Euploid	Completely Consistent
B2	+4, +16	2.16	+4, +16	
B3	+16	4.40	dup(10)(q11.21q23.33)(~52.00Mb,~54%), +16(~56%)	Partially Consistent
B4	-7, -16, -21	2.40	-7 (~69%), del(11)(p15.5p14.3)(~25.50Mb,~45%), -16 (~62%), -21(~54%)	
B5	Euploid	1.13	-15 (~63%)	Completely Inconsistent
B6	+20 (~30%)	1.88	+8 (~52%)	

BRD, Biopsy-related droplets; CNV, Copy number variation; CSL, Concentration of the sequencing library; Completely Consistent: Both TE and corresponding BRD samples were either euploid or exhibited identical CNV findings without additional discrepancies; Partially Consistent, TE and BRD samples shared at least one identical CNV, but additional non-overlapping CNVs were present in one or both samples; Completely Inconsistent, TE and BRD samples showed entirely different chromosomal findings with no overlapping CNVs.

These findings indicate that the BRDs of blastocysts contained detectable genetic material, and in our dataset, CNVs were more frequently detected in BRDs with CSL > 0.5 ng/µL, which accounted for approximately 50% of the total BRDs.

### Factors influencing the nucleic acid content in BRD

Comparisons were made between the high CSL group (HCSL, ≥ 0.5 ng/µL, 161 blastocysts) and the low CSL group (LCSL, <0.5 ng/µL, 157 blastocysts) in terms of female age, male age, duration of infertility, semen quality, total gonadotropin (GN) dosage, GN stimulation duration, number of retrieved oocytes, number of mature oocytes, number of high-quality Day3 (D3) embryos, number of formed blastocysts, number of high-quality blastocysts, developmental day of the blastocyst (D5 or D6 or D7), blastocyst grade, ICM grade, TE grade, biopsy operator, number of biopsy cells, and CNV detection results of biopsy cells (i.e., blastocyst chromosomal ploidy detected by PGT-A). The results are shown in **Table 1**.

The CSL of the BRDs was significantly correlated with the developmental day of the blastocysts (*P* = 0.018) and their chromosomal ploidy (*P* = 0.001). BRDs of D6 and D7 blastocysts had higher nucleic acid content than that of D5 blastocysts, and BRDs of non-euploid blastocysts had higher nucleic acid content than that of euploid blastocysts.

### CNV results of biopsy cells and their BRDs

The CNVs successfully detected in the 91 BRDs were compared with those detected by PGT-A, and three categories of results were observed (**Table 2**; **Figure 2**; **Supplementary Table 1**). The initial classification was defined strictly based on concordance in detailed CNV detection patterns rather than overall chromosomal ploidy. Ignoring the mosaic proportions and sizes, type I (complete consistency) accounted for 47.25% (43/91) of cases, where both samples (TE and corresponding BRD) were either euploid (B1) or showed the same CNV with no additional discrepancies (B2). Type II (partial consistency) accounted for 18.68% (17/91) of cases, where both samples shared at least one identical CNV, while additional, non-overlapping variations were also present (B3–B4). Type III (complete inconsistency) accounted for 34.07% (31/91) of the cases, including cases where CNV of one sample (TE or BRD) indicating euploidy and that of the other non-euploidy (such as B5; 18/31, 58.06%) as well as the detection of entirely different types of CNV (such as B6; 13/31, 41.94%).

From the perspective of chromosomal ploidy, the CNV results of biopsy cells and the BRDs were considered inconsistent only if one test indicated euploidy and the other indicated non-euploidy (such as B5; 18 samples in total); otherwise, they were considered consistent. In general, among the 91 samples with CNV results, the BRDs and biopsy cells showed relatively high chromosomal ploidy consistency (80.22%, 73/91).

### Association between blastocyst transfer outcomes and BRD CNV results

Among the 318 blastocysts, 158 were euploid blastocysts according to PGT-A; of these, 72 blastocysts were transferred. Among the 72 transferred blastocysts, BRD samples from 22 cases (30.56%, 22/72) were successfully sequenced and yielded CNV results (**Table 3**). Among the patients who received transferred blastocysts, 6 patients achieved live births (B7–B12), 2 had biochemical pregnancies (B13–B14), 4 had miscarriages (B15–B18), 31 were still pregnant at follow-up, and 29 were not pregnant (**Tables 3**–**5**).

**Table 3:** Transfer outcomes of 72 euploid blastocysts detected by PGT-A and testing results of their corresponding BRDs.

Transfer outcome	Number of cases						
	Total	Clinical pregnancy outcomes		CNV results			
		Clinical pregnancy	Non-clinical pregnancy	Unable to detect	Detection failed	Euploid	Non-euploid
Live Birth	6	6	—	6 100%	0 0%	0 0%	0 0%
Miscarriage	4	4	—	3 75%	0 0%	1 25%	0 0%
Biochemical Pregnancy	2	—	2	2 100%	0 0%	0 0%	0 0%
Pregnancy	31	31	—	17 54.84%	3 9.68%	10 32.26%	1 3.23%
No Pregnancy	29	—	29	18 62.07%	1 3.45%	2 6.90%	8 27.59%

BRD, Biopsy-related droplets; PGT-A, preimplantation genetic testing for aneuploidy. “—” indicates data not applicable.

**Table 4:** Association between blastocyst transfer outcomes and BRD CNV results (live birth, biochemical pregnancy, and miscarriage groups).

Blastocyst code	Female age (years)	Blastocyst developmental day	Blastocyst morphological grade	CSL	BRD CNV	Pregnancy outcome	Gestational age at miscarriage (weeks + days)	Chromosomal CMA results (amniotic fluid or POC)
B7	30	D6	4BB	Very Low	—	Live Birth	—	No Obvious Abnormalities
B8	32	D5	4BA		—	Live Birth	—	No Obvious Abnormalities
B9	38	D6	4AA		—	Live Birth	—	No Obvious Abnormalities
B10	40	D5	4BB		—	Live Birth	—	No Obvious Abnormalities
B11	26	D6	4BC		—	Live Birth	—	No Obvious Abnormalities
B12	28	D5	4BB		—	Live Birth	—	No Obvious Abnormalities
B13	39	D5	4BB		—	Biochemical Pregnancy	—	—
B14	28	D6	4BC		—	Biochemical Pregnancy	—	—
B15	39	D5	4BB	High	Euploid	Miscarriage	8^+0^	—
B16	32	D5	4BB	Very Low	—	Miscarriage	8^+3^	—
B17	32	D5	4BA		—	Miscarriage	5^+6^	—
B18	39	D6	4BC		—	Miscarriage	14^+4^	No Obvious Abnormalities

BRD, Biopsy-related droplets; CMA, Chromosomal microarray analysis; CNV, Copy number variation; CSL, Concentration of the sequencing library; POC, Products of conception. D5 indicates Day 5; D6 indicates Day 6; “Very Low” indicates CSL < 0.35 ng/μL; “High” indicates CSL ≥ 1.0 ng/μL. “—” indicates data not applicable.

**Table 5:** Association between blastocyst transfer outcomes and BRD CNV results (pregnant and non-pregnant groups).

Blastocyst code	Female age (years)	Blastocyst developmental day	Blastocyst morphological grade	BRD CNV	Pregnancy outcome
B1	32	D6	4BB	Euploid	Ongoing Pregnancy
B19	36	D6	4BB	Euploid	Ongoing Pregnancy
B20	43	D5	4BA	Euploid	Ongoing Pregnancy
B21	32	D5	4BB	Euploid	Ongoing Pregnancy
B22	41	D6	4BB	Euploid	Ongoing Pregnancy
B23	33	D6	4BB	Euploid	Ongoing Pregnancy
B24	29	D5	4BB	Euploid	Ongoing Pregnancy
B25	33	D6	4AB	Euploid	Ongoing Pregnancy
B26	33	D5	4AB	Euploid	Ongoing Pregnancy
B27	33	D5	4BB	Euploid	Ongoing Pregnancy
B28	29	D5	4BB	dup(X)(p22.33q27.3)(~140.00Mb,~39%),-Y(~32%),del(1)(q42.3q44)(~12.75Mb),del(13)(q21.33q34)(~42.17Mb,~31%)	Ongoing Pregnancy
B29	29	D6	4BB	Detection Failed	Ongoing Pregnancy
B30	26	D6	4BB	Detection Failed	Ongoing Pregnancy
B31	39	D5	4AA	Detection Failed	Ongoing Pregnancy
B32	29	D6	4BB	del(16)(q12.1q24.3)(~39.35Mb)	Not Pregnant
B33	36	D5	4BB	del(14)(q12q23.2)(~39.00Mb),-18(~31%)	Not Pregnant
B34	36	D6	3CB	-9, del(11)(q12.1q25)(~77.01Mb), del(15)(q24.1q26.3)(~27.50Mb,~52%)	Not Pregnant
B35	32	D6	4BC	+2, +4, +6, +15	Not Pregnant
B36	37	D6	4BC	dup(1)(p36.22p21.2)(~91.00Mb), dup(15)(q13.2q26.3)(~72.03Mb,~38%)	Not Pregnant
B37	29	D5	4BB	+X, -Y, dup(15)(q13.3q26.3)(~70.03Mb)	Not Pregnant
B38	32	D6	4BB	dup(17)(p13.3q24.2)(~64.50Mb,~31%)	Not Pregnant
B39	37	D6	5BB	+X, -Y, +1 (~42%), +5 (~67%), +7 (~46%), -12 (~48%), +14 (~47%), +16 (~54%), -21 (~45%)	Not Pregnant
B40	36	D6	4AB	Euploid	Not Pregnant
B41	32	D6	4BB	Euploid	Not Pregnant
B42	33	D5	4BB	Detection Failed	Not Pregnant

BRD, Biopsy-related droplets; CNV, Copy number variation. D5 indicates Day 5; D6 indicates Day 6.

Among the 6 patients with live births, the CSLs of their corresponding BRDs were all less than 0.35 ng/µL (**Tables 3**, **4**). Chromosomal microarray analysis (CMA) of the amniotic fluid revealed no chromosomal abnormalities. One (B15) of the 4 miscarriages had a BRD CSL >1.0 ng/µL, and CNV result of this BRD was euploid. Three cases (B15–B17) were early miscarriages (before 12 gestational weeks), and blastocysts of B16 and B17 belonged to the same PGT cycle from the same patient. The transfer outcome of blastocyst B18 was also very close to early miscarriage (gestational age 14^+4^ weeks), and CMA (chromosomal microarray analysis) of the villi in the postabortion products revealed no obvious chromosomal abnormalities. In general, no obvious chromosomal abnormalities were detected in the BRDs of any of the 10 blastocysts from patients with live births or miscarriage.

Among the 31 blastocysts from the pregnant patients (**Tables 3**, **5**), the CSLs of the BRDs of 17 blastocysts were too low (CSL < 0.35ng/µl) for sequencing; the BRDs of the remaining 14 blastocysts (B1, B19–B31) were subjected to CNV detection (including 3 samples with CSLs between 0.35 ng/µL and 0.5 ng/µL). Among these, results were obtained for 11 samples: 10 were euploid, and the remaining 1 sample showed non-euploidy. As of March 31, 2025, the gestational age of 24 of these 31 pregnant patients was higher than 12 weeks. Among the 29 blastocysts from non-pregnant patients (**Tables 3**, **5**), 18 had very low BRD CSLs (< 0.35 ng/µL) and did not undergo sequencing. CNVs were detected in the BRDs of the remaining 11 blastocysts (B32–B42), showing results of 8 non-euploid (i.e., aneuploid or mosaic), 2 euploid, and 1 detection failed.

Among the 72 euploid blastocysts that had been transferred (**Tables 3**, **5**), the clinical pregnancy rate for patients with euploid BRD results (11/13, 84.62%) was much higher than that of patients with non-euploid BRD results (1/9, 11.11%). In addition, CNV results of the BRDs were unavailable for 50 blastocysts (50/72, 69.44%) (**Tables 3**–**5**) due to insufficient CSL or detection failure. These blastocysts resulted in 20 pregnancies, 19 nonpregnancies, 6 live births, 3 miscarriages, and 2 biochemical pregnancies, with a clinical pregnancy rate for this type of blastocysts of 58.00% (29/50). Among the remaining 63 blastocysts with either euploid or undetermined BRD results, the overall clinical pregnancy rate was 63.49% (40/63).

In total, 72 blastocysts were transferred to 59 patients (only one blastocyst was transferred each time): 48 patients had one transfer, 10 patients had two transfers, and 1 patient had three transfers (**Table 6**). Among the 11 patients who underwent multiple transfers, 7 (P1–P7) had BRD CSLs < 0.35 ng/µL; 6 of these patients did not become pregnant after two transfers, and 1 (P7) experienced early miscarriages after both transfers. Patient P8 had three blastocyst transfers (two cycles; **Table 6** shows only the results of the two transfers in the first cycle) but did not become pregnant; the CSL of the BRD of the second blastocyst (B175) was < 0.35 ng/µL; while the BRD CNV results of the first two blastocysts indicated euploidy. Among the remaining 3 patients (P9–P11), 2 did not become pregnant after the first transfer, and the BRD CNV of their initial blastocyst transfers indicated non-euploidy; the two patients became pregnant successfully at the second transfer, and the BRD CNV of their second blastocyst transfers indicated euploidy. Patient P11 did not become pregnant after two transfers, and the BRD CNV of her transferred blastocysts both indicated non-euploidy. We compared CSL levels to explore whether concentration might be associated with embryo ploidy or clinical outcomes. Although euploid embryos generally showed lower CSL values in our cohort, this association was not absolute. Therefore, CSL should currently be regarded as an exploratory adjunct parameter, while embryo assessment remains primarily based on CNV results.

**Table 6:** BRD CNV results and clinical outcomes in patients undergoing multiple blastocyst transfers.

Patient code	Blastocyst code	Female age (years)	Duration of infertility (years)	First transfer	BRD CNV (1st)	Pregnancy outcome (1st)	Second transfer	BRD CNV (2nd)	Pregnancy outcome (2nd)
P1	B122/B124	37	3	D5, 4BB	Very Low CSL	Not Pregnant	D6, 4BB	Very Low CSL	Not Pregnant
P2	B151/B153	28	3	D5, 4AB		Not Pregnant	D5, 4AB		Not Pregnant
P3	B169/B170	34	1	D6, 4BB		Not Pregnant	D6, 4BB		Not Pregnant
P4	B178/B223	34	0	D5, 4AB		Not Pregnant	D5, 4BB		Not Pregnant
P5	B180/B181	37	1	D6, 4BB		Not Pregnant	D6, 5BB		Not Pregnant
P6	B186/B187	38	1	D5, 4BB		Not Pregnant	D5, 4BB		Not Pregnant
P7	B16/B17	32	0	D5, 4BB		Miscarriage	D5, 4BA		Miscarriage
P8	B38/B41/ B175	32	0	D5, 4AB	Euploid	Not Pregnant	D6, 4BB	Euploid	Not Pregnant
P9	B19/B40	36	2	D6, 4AB	dup(8)(p23.3p23.2)(~4.40Mb), dup(10)(q26.2q26.3)(~7.53Mb)	Not Pregnant	D6, 4BB	Euploid	Pregnant
P10	B24/B37	29	1	D5, 4BB	dup(15)(q13.3q26.3)(~70.03Mb)	Not Pregnant	D5, 4BB	Euploid	Pregnant
P11	B33/B34	36	8	D5, 4BB	del(14)(q12q23.2)(~39.00Mb), -18 (~31%)	Not Pregnant	D6, 3CB	-9, del(11)(q12.1q25)(~77.01Mb), del(15)(q24.1q26.3)(~27.50Mb,~52%)	Not Pregnant

BRD, Biopsy-related droplets; CNV, Copy number variation; CSL, Concentration of the sequencing library. D5 indicates Day 5; D6 indicates Day 6; “Very Low CSL” indicates CSL < 0.35 ng/μL. The number before the slash indicates the first transferred blastocyst, and the number after the slash indicates the second. Blastocyst numbers were renumbered according to their order of appearance in the manuscript; therefore, the numbering of the second transfer is not necessarily sequential to that of the first.

To sum up, the results for the patients who underwent multiple blastocyst transfers also revealed that the detection of non-euploidy in the BRDs was an adverse factor for successful pregnancy.

### Second biopsy results

Among the 5 blastocysts for which CNV detection failed in the first biopsy, the blastocysts were thawed with commercial thawing solution (Kitazato Corporation, Fuji, Japan) and a second biopsy was performed after blastocoel cavity re-expansion. The second biopsy was performed following the same procedures used for the first biopsy. The cells of the second biopsy were also examined for CNVs, and the BRDs obtained from the second biopsies were also analyzed as those from the first biopsies (**Table 7**, **Supplementary Table 1**).

**Table 7:** CNV consistency between second biopsy cells and corresponding first BRDs.

Blastocyst code	Blastocyst developmental stage	Blastocyst morphological grade	Second biopsy cell CNV	BRD CNV	Consistency
B2	D5	4BB	+4,+16	+4,+16	Consistent
B26	D5	4AB	Euploid	Euploid	
B61	D5	4BB	Euploid	Euploid	
B4	D6	4BC	-7, -16, -21	-7 (~69%), del(11)(p15.5p14.3)(~25.50Mb,~45%), -16 (~62%), -21 (~54%)	Partially Consistent
B107	D5	4BB	Euploid	Detection failed	—

BRD, Biopsy-related droplets; CNV, Copy number variation; D5 indicates Day 5; D6 indicates Day 6. “—” indicates data not applicable.

BRDs from the second biopsies had very low CSLs (< 0.3 ng/µL), making CNV sequencing unfeasible. Among the BRDs collected from the first biopsy, the CSLs of four were high (> 1.0 ng/µL), and that of the remaining one was low (but greater than 0.5 ng/µL). All these 5 samples were subjected to sequencing analysis. CNVs of BRDs of two blastocysts (B26 and B61) indicated euploidy, while that of blastocyst B2 showed +4,+16; all the three matched the CNV results of the biopsy cells from the second biopsy. For blastocyst B4, the results of cells from the second biopsy were essentially consistent with that of the BRD from the first biopsy. On the other hand, the results of biopsy cells and BRD for blastocyst B107 could not be compared because of sequencing failure with BRD from the first biopsy.

Among the 5 blastocysts for which CNV detection in the first biopsy failed, the BRD samples from the first biopsy and the cells from the second biopsy showed relatively high consistency (80%, 4/5) in chromosomal ploidy.

## Discussion

At present, PGT depends mainly on biopsy. In addition to biopsy cells for genetic testing, biopsy-related droplets (BRDs), which are byproducts of the procedure, can also be collected. There are currently no studies on whether these droplets contain detectable genetic material, nor on the characteristics and potential clinical applications of such material. Supplementary detection was integrated with conventional detection in this study. By collecting and performing genetic analysis on BRDs, our study aimed to address the limitation of conventional method, such as in decisions regarding repeat biopsies, in comprehensive determination of embryo chromosomal ploidy and in accurate assessment of mosaic embryos. Furthermore, this study explored the clinical potential and guiding principles of these auxiliary detection methods in these areas.

### Practical foundations of BRD-based genetic testing

Collecting trophectoderm cells through biopsy for NGS analysis is the current mainstream method for PGT. Recent studies have shown that blastocoel fluid (BF) and spent culture medium (SCM) contain detectable genetic material (13–16), indicating that they have great potential for noninvasive genetic testing. However, the biological origin and release mechanism of these genetic materials into the culture medium remain unclear. (17–21). The unclear mechanisms and challenges in detecting trace-level genetic materials in these noninvasive samples have largely limited the clinical application of this technique. At present, noninvasive genetic analysis of embryos is still in its exploratory stage for both scientific research and clinical application, and thus it cannot yet replace conventional invasive methods. Some studies have reported that combining noninvasive genetic testing with morphological grading can provide a highly accurate assessment of the developmental potential of embryos, offering a promising approach (11, 17, 22, 23).

The collection of SCM requires strict procedures, including single-droplet culture of individual blastocyst and additional removal of granulosa cells (11). In addition, the use of SCM may face problems related to poor timeliness and susceptibility to external interference (such as maternal contamination) (10, 13, 19). As the blastocoele is a relatively independent and closed internal structure of the blastocyst, the use of BF for detection can ensure good timeliness and purity. However, BF needs to be effectively obtained via blastocoel aspiration (16, 18, 24), a minimally invasive method, thus limiting its widespread clinical use. At present, the combination of SCM and BF released by laser-assisted shrinkage, has been explored as an approach to enhance the content of cell-free DNA (cfDNA) in noninvasive testing (11, 19). However, laser-assisted shrinkage has low efficiency, and the degree of shrinkage varies between individuals, making effective collection of BF difficult (25). However, in PGT, blastocyst biopsy is a routine procedure, and the invasive opening and washing processes allow the effective collection of BF. Therefore, additional assisted hatching or blastocoelic fluid aspiration is not essential for BRD analysis.

This study revealed that more than 50% of the BRDs contained detectable genetic material. These findings suggested that the biopsy procedure releases a measurable amount of BF into the droplets. In this context, BF DNA levels correlate with apoptotic gene expression and chromosomal abnormality status, which is consistent with a potential contribution from apoptotic or abnormal cells to the genetic material within the blastocoel cavity (26). Genetic material in BF/SCM may vary with developmental timing, and higher DNA yields have been reported in later-stage embryos in some noninvasive testing studies. Thus, D6 and D7 blastocysts contained higher concentrations of genetic material than D5 blastocysts, and BRDs from non-euploid blastocysts showed higher CSL values than those from euploid blastocysts in our cohort. While apoptosis has been proposed as one potential contributor to blastocoel cfDNA, evidence remains largely correlative. This finding is consistent with the results of previous studies on noninvasive genetic testing (10, 14, 27). The blastocyst morphological grade did not affect the concentration or detection results of noninvasive genetic material (10, 16), consistent with our study. BRD samples from the HCSL and LCSL groups were not significantly different in terms of the overall grade, ICM grade or TE grade of the blastocysts. Many studies have identified cell apoptosis as a key source of noninvasive detection samples (19, 20), but it is not the sole source. In general, although low-quality blastocysts may have a higher likelihood of non-euploidy (28–30), they also have fewer cells and lower metabolic efficiency, which also affects the release of genetic material into the BF or SCM. Katharine et al. reported that cfDNA detection rate was lower in low-quality groups of frozen-thawed blastocysts cultured for varying duration (19).

Previous studies have shown that the concordance rate between noninvasive detection and TE detection in terms of chromosomal ploidy is 30%–100% (8, 13, 14, 16, 20, 31). In this study, BRDs with detectable CNVs and their corresponding biopsy cells had an 80.22% (73/91) concordance rate of chromosomal ploidy. In addition, a proportion of blastocysts are mosaic (10–20%) (32), which are difficult to identify through biopsy-based detection methods (10, 33). In this context, BRDs may provide complementary information in assessing blastocyst chromosomal ploidy.

For noninvasive samples with low cfDNA concentrations or failed detection of CNV, a number of studies have shown that the corresponding embryos have favorable pregnancy outcomes upon transfer (although lower than those of embryos detected as euploid by the corresponding noninvasive samples) (17, 23), consistent with the finding (58.00%) of this study. The biological origin of embryo-derived cfDNA remains incompletely understood and is likely multifactorial. Therefore, it would be inappropriate to infer that euploid embryos inherently release less cfDNA or have reduced amplification performance. Apoptosis has been proposed as one potential contributor to embryo-derived cfDNA; however, available evidence is largely correlative and cfDNA likely originates from multiple mechanisms (20, 34, 35). Notably, some samples with high cfDNA concentrations still failed in CNV detection, possibly due to unidentified underlying mechanisms (10, 20). Given the extremely low cfDNA content and fragmented nature of noninvasive samples (18, 19), as well as the unknown factors introduced by unidentified sources, it may be premature to use these samples for precise clinical diagnostics. Nevertheless, preliminary detection of abnormal chromosomal signals in noninvasive samples is sufficient for application as a supplementary analytical method; some studies have even relied solely on the cfDNA concentration to guide the transfer priority of fresh blastocysts (36).

Although BRD-based testing is primarily considered as an auxiliary method, its protocols for sample collection, amplification, detection, and analysis still require further optimization. For example, several strategies should be used to overcome the maternal contamination: additional blastocyst degranulation prior to biopsy, replacing the biopsy droplet after laser opening of the zona pellucida, omitting the collection of WDs, skipping the lysis step before amplification, and incorporating maternal contamination correction into the sequencing data analysis. These combined measures can significantly reduce maternal contamination.

### Assisted selection of actual euploid blastocysts

Recent studies have shown that embryo mosaicism may be more prevalent than initially believed (9, 37), while the authenticity of the results labeling embryos as euploid by PGT-A is rarely questioned (8, 9, 20). In this study, of the 72 transferred blastocysts (identified as euploid based on PGT-A, **Table 3**), BRD analysis detected aneuploidy or mosaicism in 9 blastocysts (12.50%, 9/72), 8 of which (88.89%, 8/9) failed to result in pregnancy. This result is surprising and may be due to the small sample size. Among the remaining 63 blastocysts, which were either detected as euploid or with undetermined chromosomal status based on BRD analysis (including 6 blastocysts that resulted in live births, 4 in miscarriages, 30 in ongoing pregnancies, 21 in pregnancy failure, and 2 in biochemical pregnancies), the clinical pregnancy rate was 63.49% (40/63). Specifically, the clinical pregnancy rate for blastocysts detected as euploid by BRD analysis (84.62%, 11/13) was higher than that for those with failed BRD analysis (58.00%, 29/50), which is consistent with findings of relevant studies based on SCM and BF (14). We compared blastocysts with different CSL levels to explore whether CSL might reflect embryo ploidy or clinical outcome. Although euploid embryos generally showed lower CSL values, this association was not absolute, and some euploid embryos with higher CSL still achieved favorable pregnancy outcomes. Therefore, CSL currently serves only as an exploratory adjunct parameter, while embryo assessment remains primarily based on CNV results.

Therefore, BRD-based genetic analysis may provide additional information that may potentially help in avoiding the preferential transfer of blastocysts that are suspected to be mosaic, which are often associated with reduced pregnancy potential. For instance, in patient P9 and P10 (**Table 6**), the outcomes might have been different if blastocyst 2 (B40 and B37), with slightly poorer morphology, had been prioritized for transfer instead of blastocyst 1 (B19 and B24), which, despite having the best morphology, was likely mosaic according to BRD analysis. Nevertheless, this observation is exploratory and requires validation in larger cohorts.

### Assisting in investigating the causes of transfer failure

Although the average clinical pregnancy rate of 56.94% (41/72) in our study was not high (**Table 3**), it was comparable to previous reports in similar populations (22, 31). Considering potential complications such as miscarriage, the final live birth rate is expected to be even lower (likely below 50%). However, this is typical for PGT-A patients, who often present with advanced maternal age, recurrent spontaneous abortion, or repeated implantation failure. In addition to embryonic factors, various non-embryonic conditions can also affect clinical pregnancy outcomes. Indeed, as noted in Section 4.2, of the 29 blastocysts that did not result in pregnancy, 17 were from patients who had experienced at least 2 prior transfer failures (**Table 6**). These findings strongly suggest that recurrent pregnancy failure in these patients may be driven by factors beyond embryo quality. In patients who have experienced recurrent transfer failures, thawing and analyzing the BRD from the first transferred blastocyst may help determine whether the failure was truly due to embryonic factors or potentially overlooked maternal conditions that were initially attributed to chance. Such information would provide a more reliable basis for identifying non-embryonic factors and adjusting accordingly, helping to prevent unnecessary embryo transfers and wastage.

When pregnancy does not occur after euploid embryo transfer, patients often undergo extensive clinical investigations or protocol adjustments driven by anxiety (22). However, the possibility of false-negative results in PGT-A is rarely considered. For patients P9-P11 (**Table 6**), the combination of outcomes of multiple transfers and of BRD genetic testing revealed that the cause of transfer failure was more likely related to embryonic factors, making further investigations or adjustments unnecessary. Therefore, improved accuracy in embryo chromosomal ploidy detection—particularly through BRD analysis—can be crucial in identifying the true cause of transfer failure and guiding more informed clinical actions.

### Assessment of second biopsy necessity

The PGT test yields a proportion (1%-2%) of test failures or suspected abnormal results (7), typically due to factors such as sample quality, biopsy operation, and genetic testing. Given the high cost of PGT and the preciousness of the blastocysts, if a second biopsy is required to confirm the diagnosis, the frozen blastocyst needs to be thawed first and then refrozen after the biopsy cells is collected again for the second biopsy. This additional operation may have an adverse effect on the developmental potential of the blastocyst (7), while the patient will have to bear the high costs of the second biopsy. Currently, there is a lack of simple and effective auxiliary means to provide evidence for deciding to perform a second biopsy, that is, to assess the potential benefit of a second biopsy at the technological level.

Noninvasive examination materials have been mentioned in relevant studies as backup samples in cases of initial biopsy failure (10, 20), but owing to the low incidence of such failures, these studies did not provide specific practical data for clinical use. In this study, for three blastocysts (B2, B26 and B61) that underwent a second biopsy, the CNV results from BRD samples collected in the first biopsy were fully concordant with the TE results obtained from the second biopsy (**Table 7**), suggesting a high likelihood of cell transfer failure or incomplete transfer during the first biopsy. The preliminary findings from our study suggest that BRD-based genetic analysis may serve as a useful reference for determining whether a second biopsy is warranted. However, these observations should be interpreted with caution and warrant validation in studies with larger sample sizes.

### Potential application in the assessment of mosaic blastocysts

Today, attitudes toward mosaic blastocysts, especially blastocysts with low mosaic proportions (< 50%), are generally lenient and more accepting than in the past (38, 39, Reproductive Medicine Society Of The Chinese Medical Doctor Association, 2024). However, it is well established that mosaic blastocysts are associated with lower pregnancy rates and higher miscarriage rates. Generally, a higher level of mosaicism correlates with decreased implantation potential and increased risk of adverse outcomes (38–40). Blastocysts with lower mosaicism levels are more likely to undergo self-repair and return to a euploid state, similar to the pathogenic threshold for mitochondrial diseases, where disease exhibits only when abnormal mitochondria exceed a certain proportion (41). Given the limitations of PGT-A, the measured mosaicism and its ratio may not reflect the true chromosomal composition of the blastocyst, highlighting the need to integrate more representative tools such as BRD-based genetic analysis. Mosaic blastocysts should neither be discarded indiscriminately, nor should they be blindly transferred, risking pregnancy failure. The detection of mosaicism is affected by the sampling location and testing process, both of which can result in nonrepresentative results and false positives.

In this study, 43 mosaic blastocysts were identified by PGT-A, accounting for 13.52% (43/318) of all blastocysts, and 22 of them (51.16%) were classified as HCSL (≥ 0.5 ng/µL) according to BRD analysis. Compared with 72 euploid blastocysts (transplanted) detected by PGT-A (**Tables 3**–**5**), mosaic blastocysts showed a lower proportion of euploid BRD results (18.06% vs. 11.63%) and also a lower proportion of LCSL or detection failure (69.44% vs. 58.14%). In contrast, the proportion of non-euploidy detected by BRD analysis was more than twice as high in mosaic blastocysts (30.23%) compared to euploid blastocysts (12.50%). It is plausible to speculate that mosaic blastocysts identified as non-euploid by BRD analysis (30.23%, 13/43) may have a higher probability of adverse pregnancy outcomes compared with those identified as euploid by BRD analysis, based on the data from euploid blastocysts presented in sections 3.4 and 4.2.

No significant chromosome abnormalities were detected in the BRDs of up to 69.77% (30/43) of the mosaic blastocysts. The pregnancy outcomes of transferring such blastocysts remain unclear and therefore merits further investigation. In the study by Xinyuan et al., the results of donated blastocysts were used as the gold standard, and up to 85.4% of the previously identified mosaic blastocysts were classified as euploid upon redetection (42). Unfortunately, owing to institutional policy and patient preferences, our center seldom transfers mosaic embryos, and thus is unable to provide follow-up pregnancy outcome data for verification.

### General application principles and limitations of BRD-based genetic analysis

Currently, the clinical utility of noninvasive genetic testing for diagnostic purposes remains limited and requires further rigorous research and validation. However, when used as an auxiliary tool alongside PGT-A, it offers a more robust and feasible approach. This study employs BRD-based genetic analysis as an adjunct to conventional testing, aiming to provide a more accurate representation of the embryo’s true overall chromosomal status, thereby optimizing clinical outcomes. This method does not provide a definitive ‘yes or no’ judgment on the embryo’s fate but rather offers recommendations regarding the embryo’s priority for transfer (17, 22, 23).

**Figure 3:**
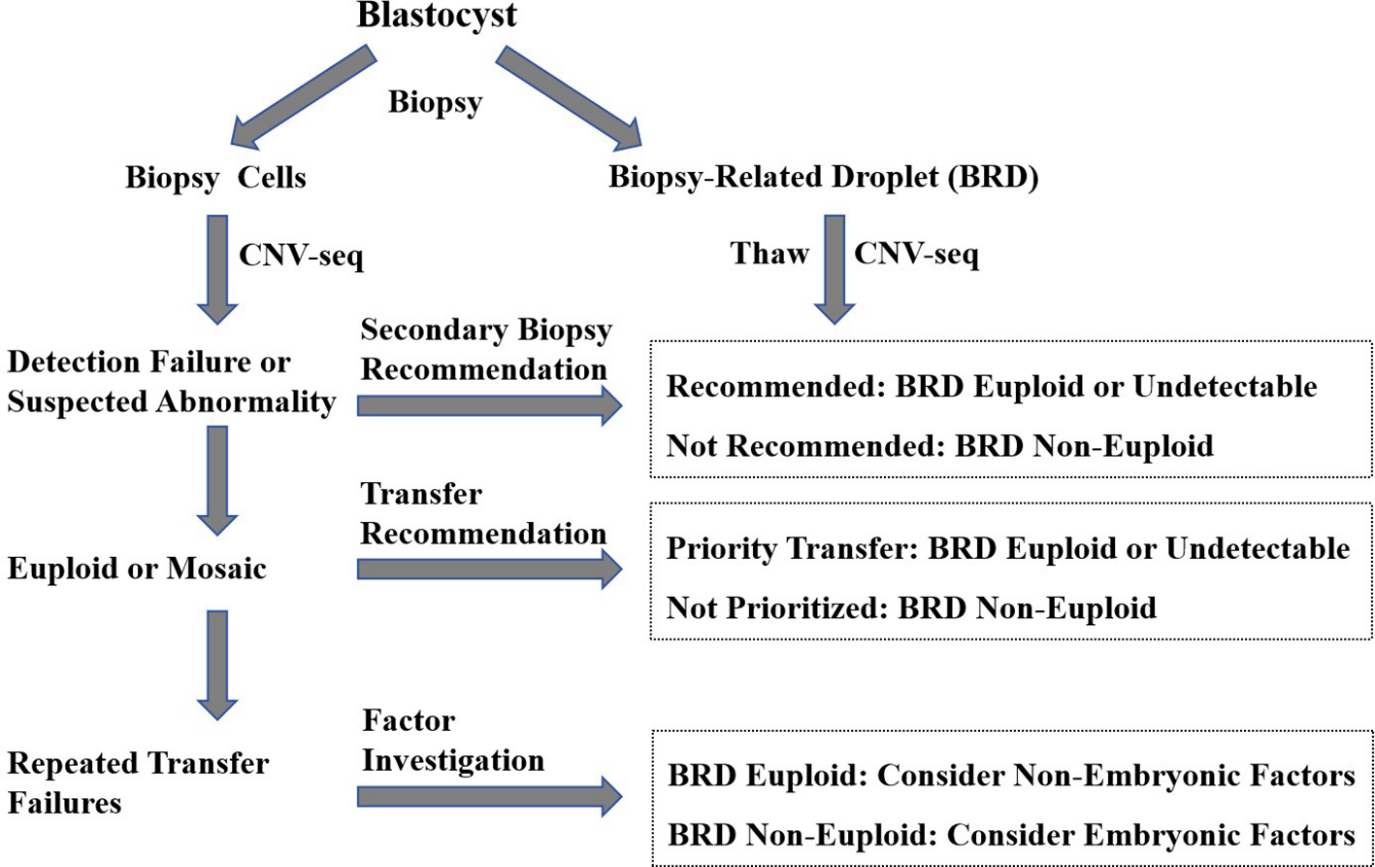
The specific process and principles for the application of BRD-based genetic analysis. This flowchart outlines the process for utilizing BRD in decision-making for secondary biopsy recommendations, transfer prioritization for euploid (or mosaic) blastocysts identified by PGT-A, as well as considerations for repeated transfer failures.

Based on our findings, we do not routinely recommend BRD-based analysis for all blastocysts. Instead, BRDs are selectively thawed and analyzed when clinically relevant, to assist in embryo evaluation and transfer decisions. The specific process and principles (**Figure 3**) for the application of this technique are as follows: (1) In the event of detection failure, if the BRD analysis indicates non-euploidy, a second biopsy is not recommended. (2) For euploid blastocysts identified by PGT-A, if BRD analysis indicates euploidy, that blastocyst should be prioritized for transfer. If their chromosomal ploidy cannot be determined by BRD analysis, that blastocyst has secondary transfer priority; If BRD analysis indicates non-euploidy, the blastocyst should not be prioritized for transfer. Mosaic blastocysts can be treated with the same considerations. (3) In patients who have experienced unsuccessful outcomes after repeated transfers of euploid embryos, if the BRD analysis indicates euploidy, the cause is more likely non-embryonic; if non-euploidy, the failure is more likely embryo-related.

This study has several limitations. The BRDs used in this study may also contain embryonic cells, cellular debris introduced by the biopsy procedure, as well as other genetic contaminants, such as granulosa cells; therefore, protocols for sample collection, amplification, sequencing, and analysis need to be further optimized. In addition, this was a single-center study in which the research objects were embryos from PGT-A patients. Some subgroup analyses were based on limited sample sizes, thus the relevant conclusions require more extensive verification.

## Conclusion

By collecting the byproducts of biopsy (i.e., BRD) and performing genetic analysis on this material, BRD-based analysis may serve as a complementary source of information to conventional PGT-A. The results showed that this method shows promise in aiding the decision to perform a second biopsy and in assessing the implantation potential of euploid blastocysts. BRD-based genetic analysis can serve as a valuable complement to PGT-A, offering additional insights.

## Data Availability

The original contributions presented in the study are included in the article/**Supplementary Material**. Further inquiries can be directed to the corresponding authors.
