# Supplementary material for: Genetic analysis of biopsy-related droplets in patients undergoing PGT-A and its potential application
Source: Front Endocrinol (Lausanne). 2026 May 4;17:1734617. doi: 10.3389/fendo.2026.1734617 (PMC13180553; doi:10.3389/fendo.2026.1734617)
Supplement: Supplementary file 1 [file Table1.docx]

**Supplementary Table 1** CNV Results of Biopsy Cells and Their Corresponding BRDs

| Blastocyst code | Developmental Day | Morphological Grade | BRD CNV | Consistency |
| --- | --- | --- | --- | --- |
| B1 | D6 | 4BB | Biopsy Cells: Euploid | 2 |
|  |  |  | 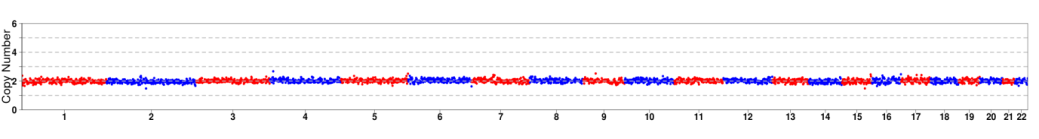 |  |
|  |  |  | BRD: Euploid |  |
|  |  |  | 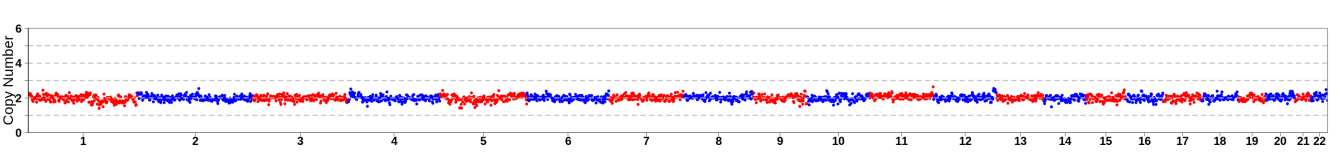 |  |
| B2 | D5 | 4BB | Biopsy Cells: +4,+16 | 2 |
|  |  |  | 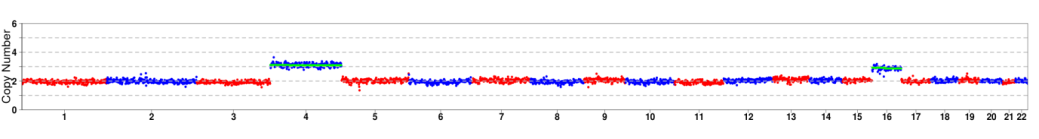 |  |
|  |  |  | BRD: +4,+16 |  |
|  |  |  | 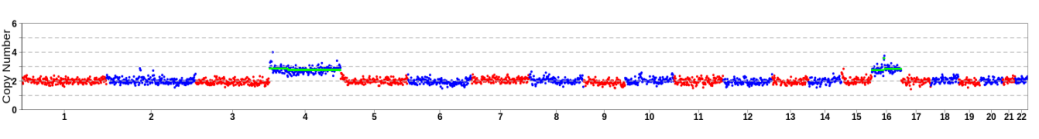 |  |
| B3 | D6 | 4BB | Biopsy Cells: +16 | 1 |
|  |  |  | 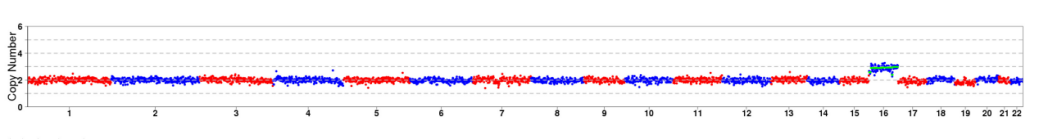 |  |
|  |  |  | BRD: dup(10)(q11.21q23.33)(~52.00Mb,~54%),+16(~56%) |  |
|  |  |  | 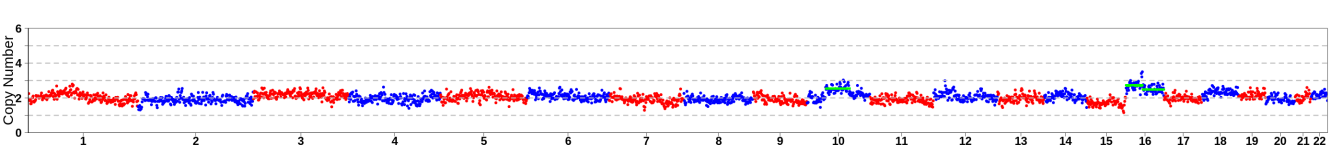 |  |
| B4 | D6 | 4BC | Biopsy Cells: -7,-16,-21 | 1 |
|  |  |  | 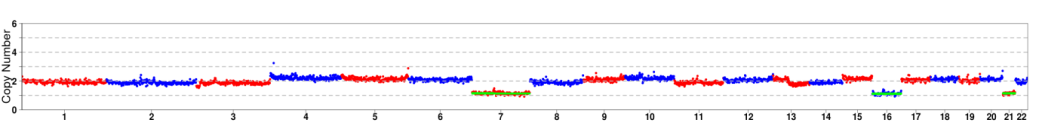 |  |
|  |  |  | BRD: -7(~69%),del(11)(p15.5p14.3)(~25.50Mb,~45%),-16(~62%),-21(~54%) |  |
|  |  |  | 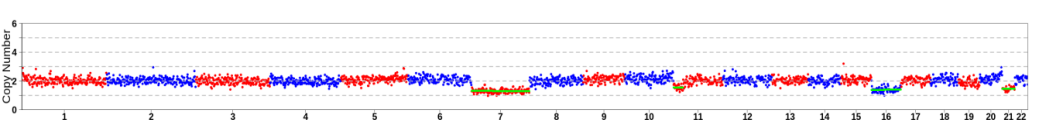 |  |
| B5 | D6 | 4BA | Biopsy Cells: Euploid | 0 |
|  |  |  | 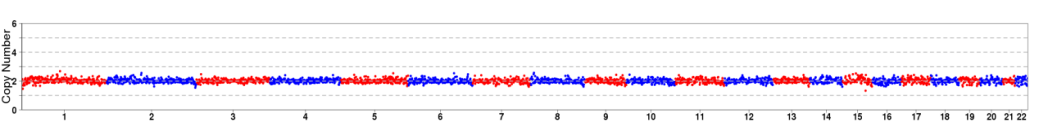 |  |
|  |  |  | BRD: -15(~63%) |  |
|  |  |  | 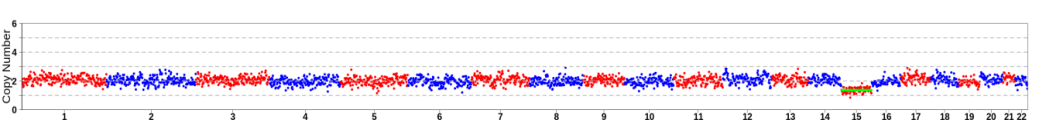 |  |
| B6 | D5 | 4BC | Biopsy Cells: +20(~30%) | 0 |
|  |  |  | 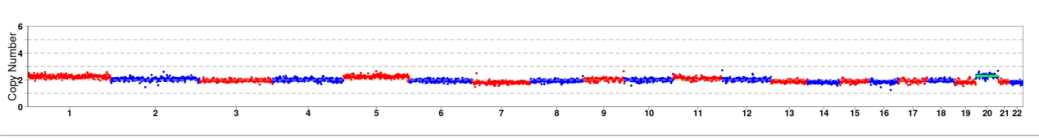 |  |
|  |  |  | BRD: +8(~52%) |  |
|  |  |  | 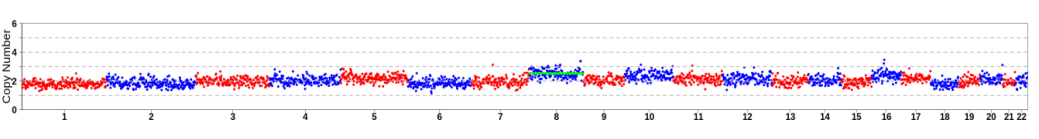 |  |
| B15 | D5 | 4BB | Biopsy Cells: Euploid | 2 |
|  |  |  | 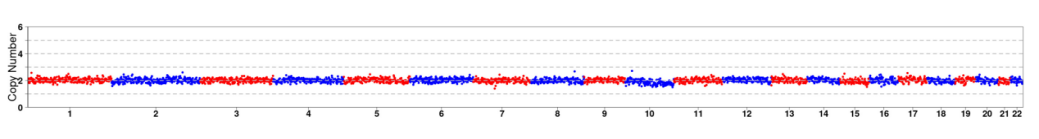 |  |
|  |  |  | BRD: Euploid |  |
|  |  |  | 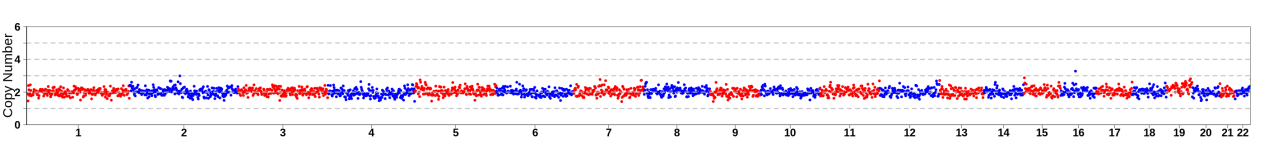 |  |
| B19 | D6 | 4BB | Biopsy Cells: Euploid | 2 |
|  |  |  | 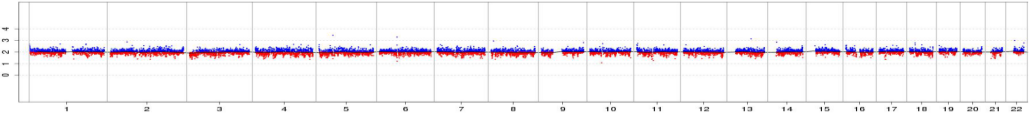 |  |
|  |  |  | BRD: Euploid |  |
|  |  |  | 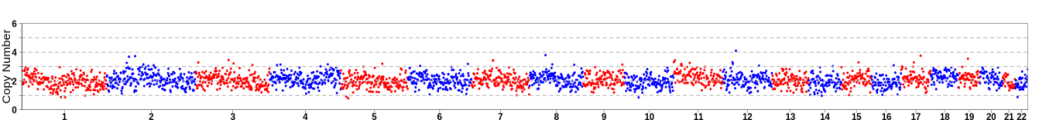 |  |
| B20 | D5 | 4BA | Biopsy Cells: Euploid | 2 |
|  |  |  | 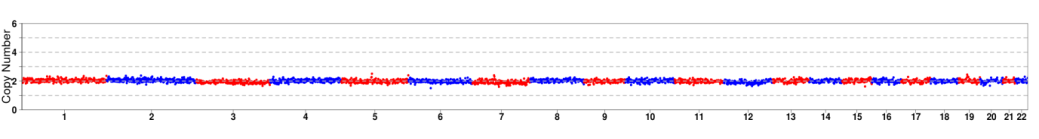 |  |
|  |  |  | BRD: Euploid |  |
|  |  |  | 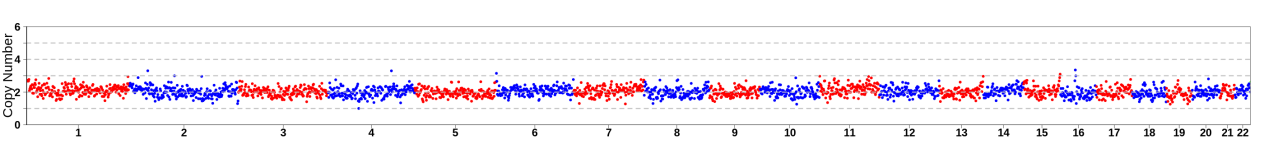 |  |
| B21 | D5 | 4BB | Biopsy Cells: Euploid | 2 |
|  |  |  | 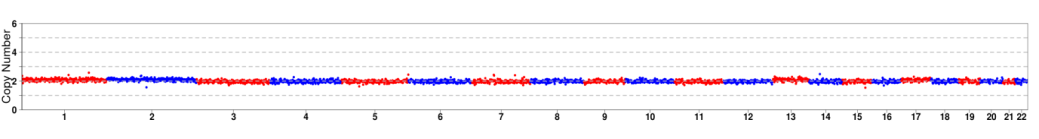 |  |
|  |  |  | BRD: Euploid |  |
|  |  |  | 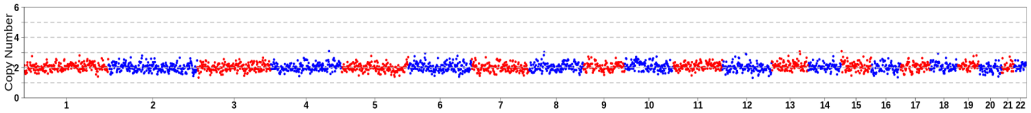 |  |
| B22 | D6 | 4BB | Biopsy Cells: Euploid | 2 |
|  |  |  | 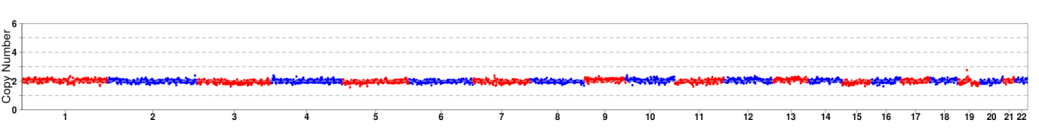 |  |
|  |  |  | BRD: Euploid |  |
|  |  |  | 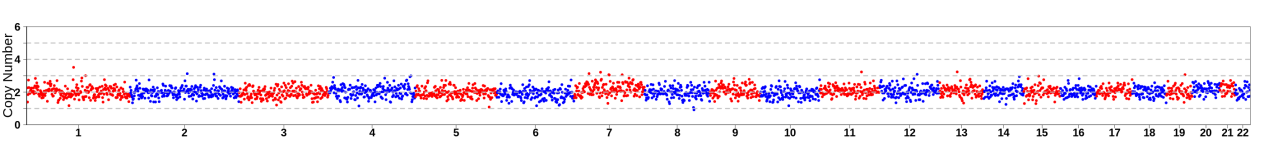 |  |
| B23 | D6 | 4BB | Biopsy Cells: Euploid | 2 |
|  |  |  | 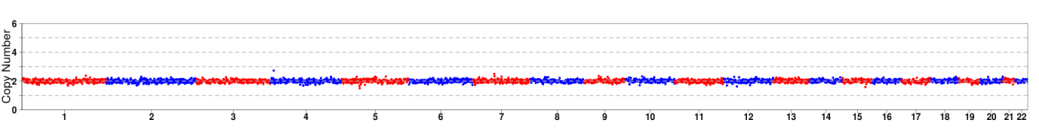 |  |
|  |  |  | BRD: Euploid |  |
|  |  |  | 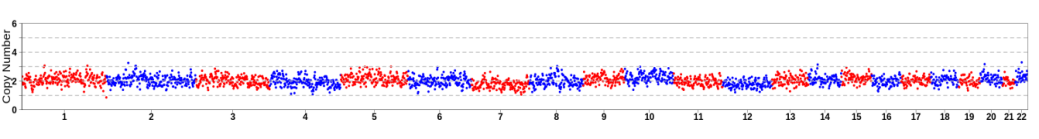 |  |
| B24 | D5 | 4BB | Biopsy Cells: Euploid | 2 |
|  |  |  | 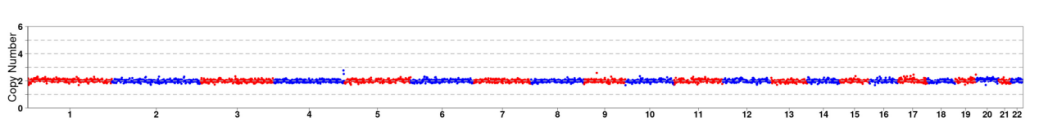 |  |
|  |  |  | BRD: Euploid |  |
|  |  |  | 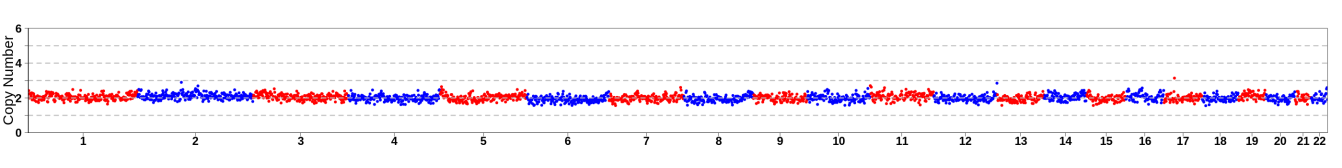 |  |
| B25 | D6 | 4AB | Biopsy Cells: Euploid | 2 |
|  |  |  | 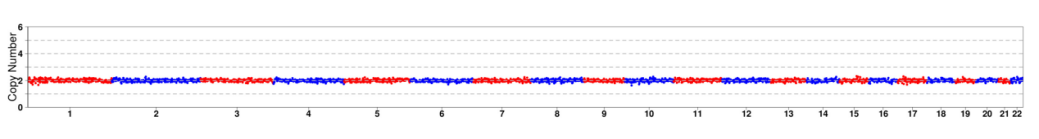 |  |
|  |  |  | BRD: Euploid |  |
|  |  |  | 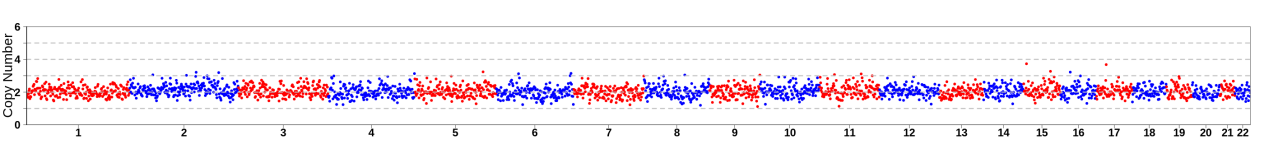 |  |
| B26 | D5 | 4AB | Biopsy Cells: Euploid | 2 |
|  |  |  | 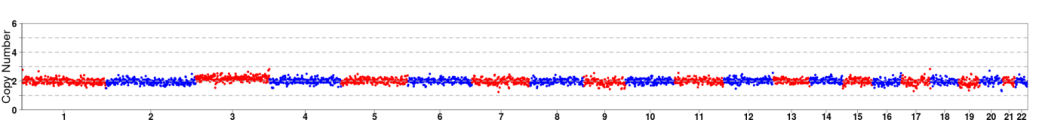 |  |
|  |  |  | BRD: Euploid |  |
|  |  |  | 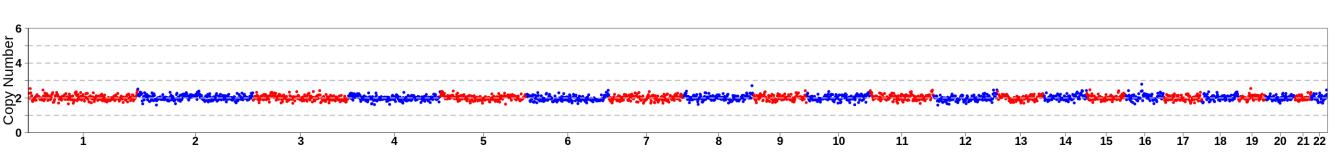 |  |
| B27 | D5 | 4BB | Biopsy Cells: Euploid | 2 |
|  |  |  | 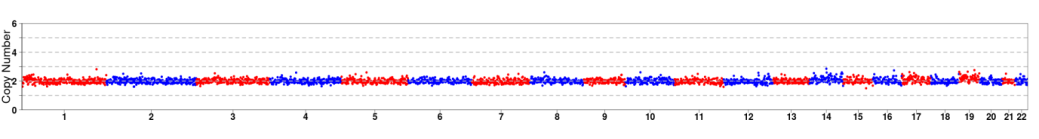 |  |
|  |  |  | BRD: Euploid |  |
|  |  |  | 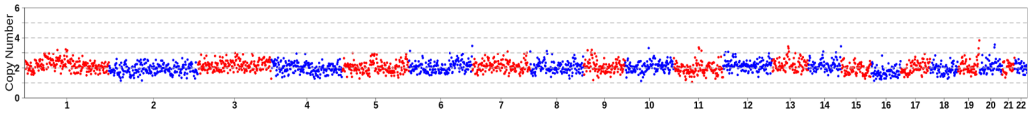 |  |
| B28 | D5 | 4BB+ | Biopsy Cells: Euploid | — |
|  |  |  | 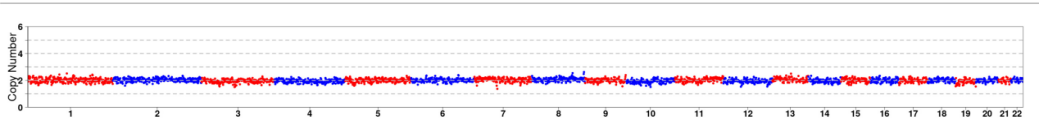 |  |
|  |  |  | BRD: dup(X)(p22.33q27.3)(~140.00Mb,~39%),-Y(~32%),del(1)(q42.3q44)(~12.75Mb),del(13)(q21.33q34)(~42.17Mb,~31%) |  |
|  |  |  | 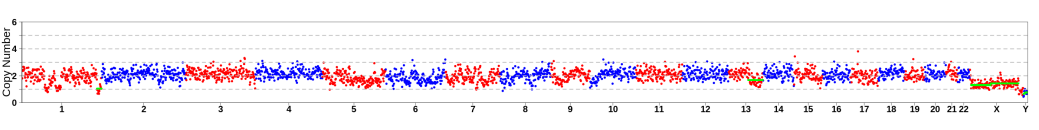 |  |
| B29 | D6 | 4BB | Biopsy Cells: Euploid | — |
|  |  |  | 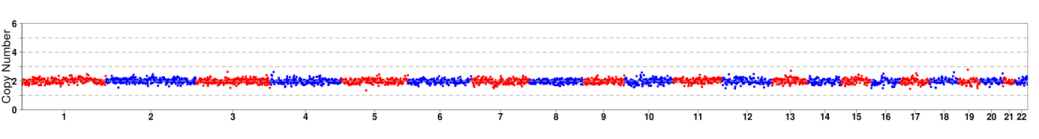 |  |
|  |  |  | BRD: Failed Detection |  |
|  |  |  | 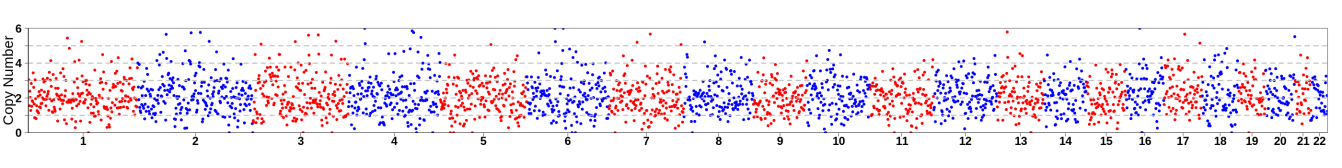 |  |
| B30 | D6 | 4BB | Biopsy Cells: Euploid | — |
|  |  |  | 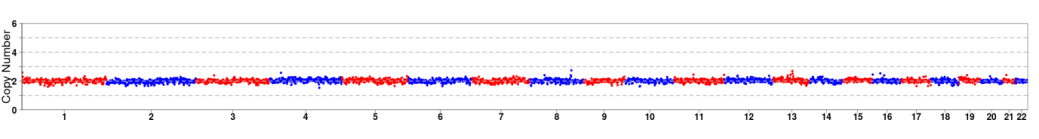 |  |
|  |  |  | BRD: Failed Detection |  |
|  |  |  | 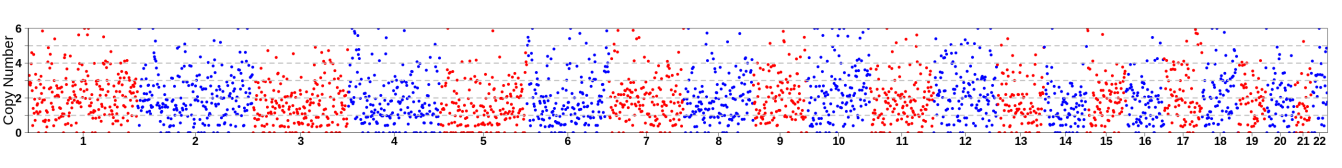 |  |
| B31 | D5 | 4AA | Biopsy Cells: Euploid | — |
|  |  |  | 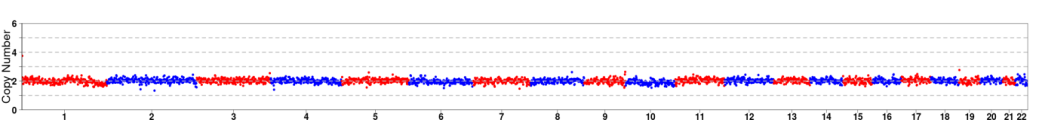 |  |
|  |  |  | BRD: Failed Detection |  |
|  |  |  | 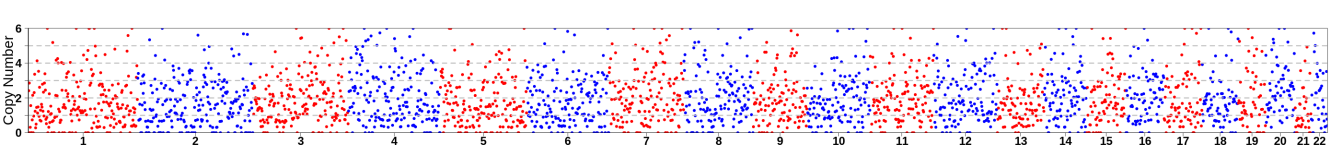 |  |
| B32 | D6 | 4BB | Biopsy Cells: Euploid | 0 |
|  |  |  | 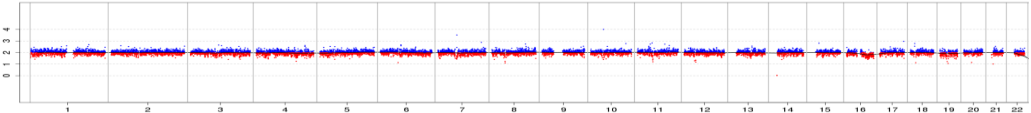 |  |
|  |  |  | BRD: del(16)(q12.1q24.3)(~39.35Mb) |  |
|  |  |  | 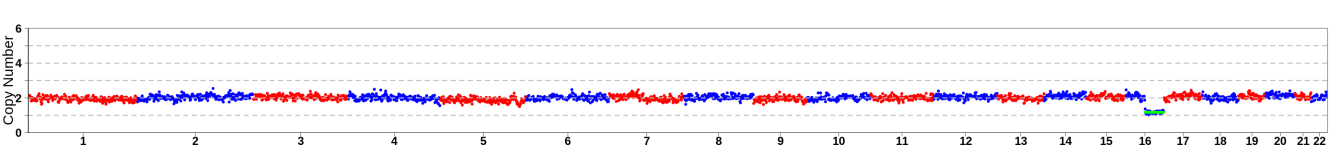 |  |
| B33 | D5 | 4BB | Biopsy Cells: Euploid | 0 |
|  |  |  | 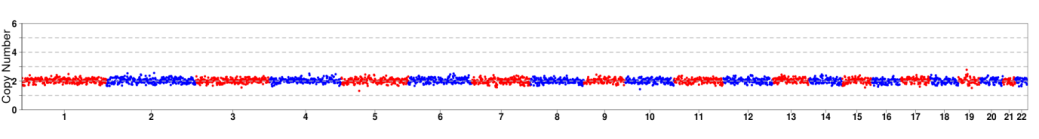 |  |
|  |  |  | BRD: del(14)(q12q23.2)(~39.00Mb),-18(~31%) |  |
|  |  |  | 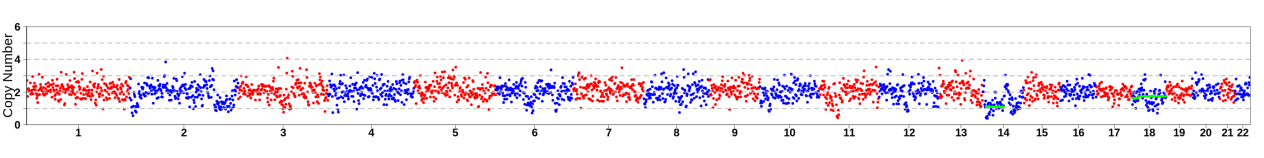 |  |
| B34 | D6 | 3CB | Biopsy Cells: Euploid | 0 |
|  |  |  | 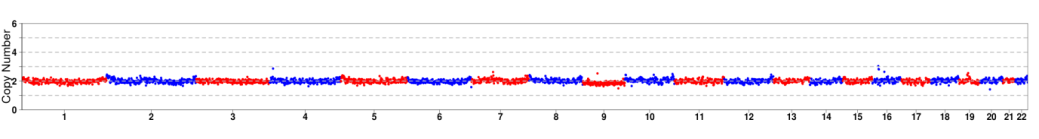 |  |
|  |  |  | BRD: -9,del(11)(q12.1q25)(~77.01Mb),del(15)(q24.1q26.3)(~27.50Mb,~52%) |  |
|  |  |  | 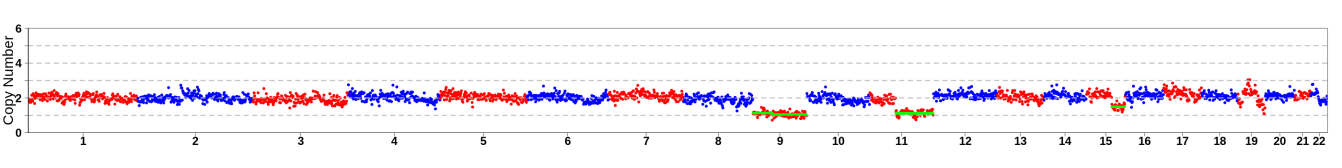 |  |
| B35 | D6 | 4BC | Biopsy Cells: Euploid | 0 |
|  |  |  | 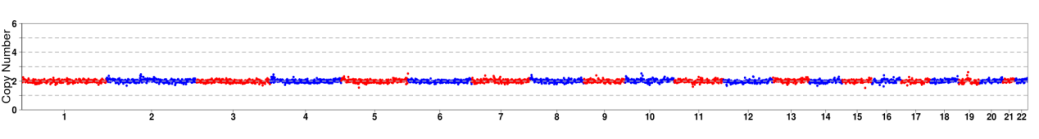 |  |
|  |  |  | BRD: +2,+4,+6,+15 |  |
|  |  |  | 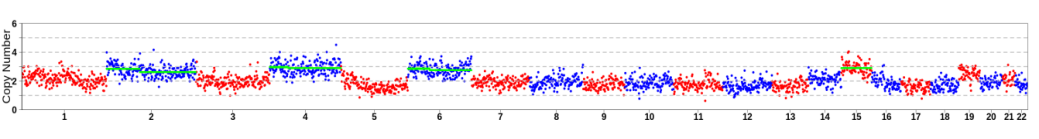 |  |
| B36 | D6 | 4BC | Biopsy Cells: Euploid | 0 |
|  |  |  | 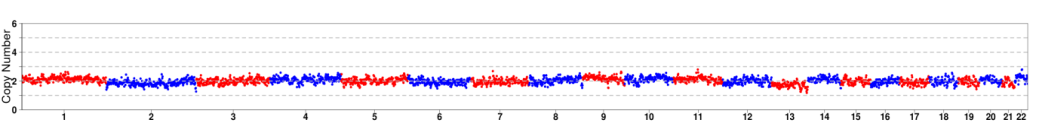 |  |
|  |  |  | BRD: dup(1)(p36.22p21.2)(~91.00Mb),dup(15)(q13.2q26.3)(~72.03Mb,~38%) |  |
|  |  |  | 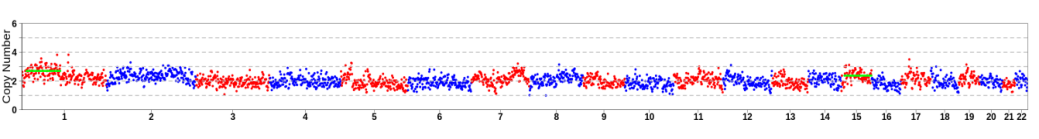 |  |
| B37 | D5 | 4BB | Biopsy Cells: Euploid | 0 |
|  |  |  | 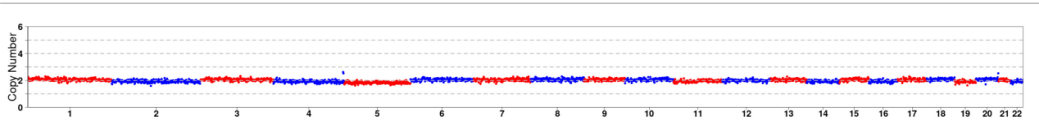 |  |
|  |  |  | BRD: +X,-Y,dup(15)(q13.3q26.3)(~70.03Mb) |  |
|  |  |  | 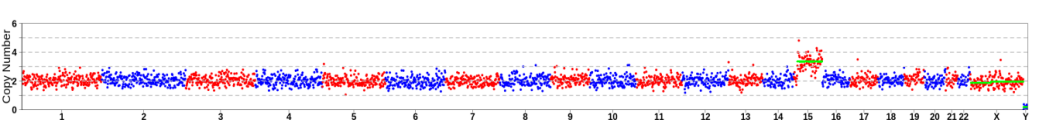 |  |
| B38 | D6 | 4BB | Biopsy Cells: Euploid | 0 |
|  |  |  | 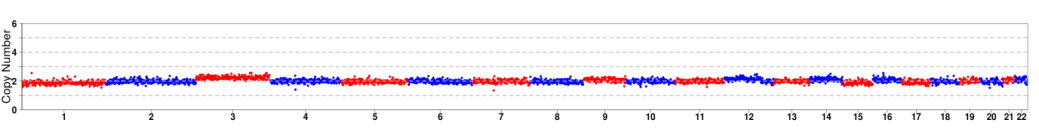 |  |
|  |  |  | BRD: dup(17)(p13.3q24.2)(~64.50Mb,~31%) |  |
|  |  |  | 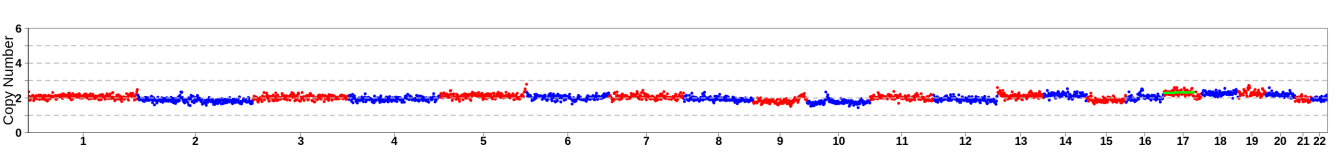 |  |
| B39 | D6 | 5BB | Biopsy Cells: Euploid | 0 |
|  |  |  | 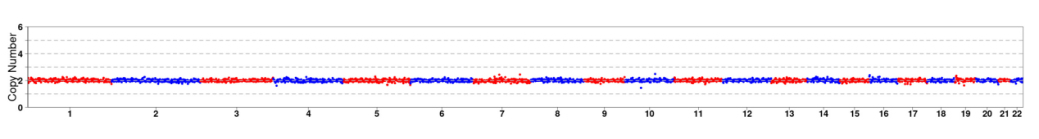 |  |
|  |  |  | BRD: +X,-Y,+1(~42%),+5(~67%),+7(~46%),-12(~48%),+14(~47%),+16(~54%),-21(~45%) |  |
|  |  |  | 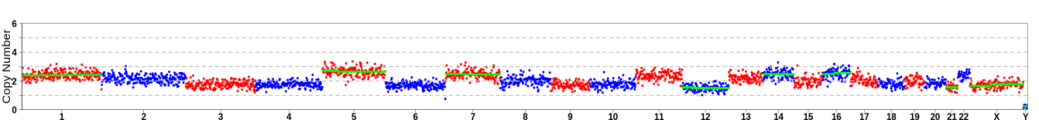 |  |
| B40 | D6 | 4AB | Biopsy Cells: Euploid | 2 |
|  |  |  | 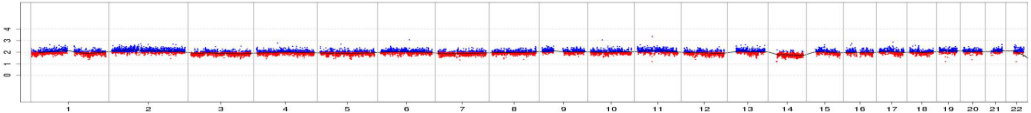 |  |
|  |  |  | BRD: Euploid |  |
|  |  |  | 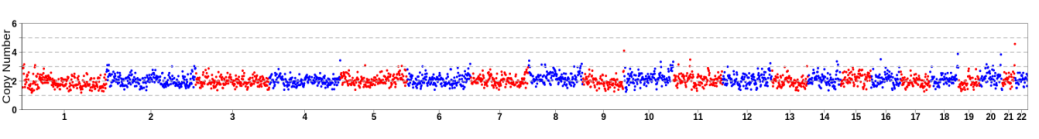 |  |
| B41 | D6 | 4BB | Biopsy Cells: Euploid | 2 |
|  |  |  | 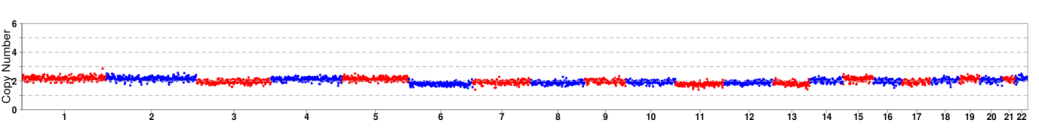 |  |
|  |  |  | BRD: Euploid |  |
|  |  |  | 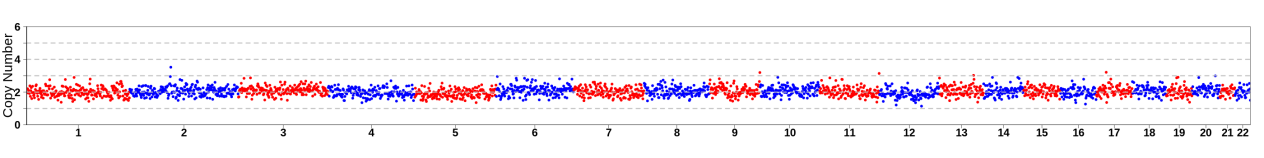 |  |
| B42 | D5 | 4BB | Biopsy Cells: Euploid | — |
|  |  |  | 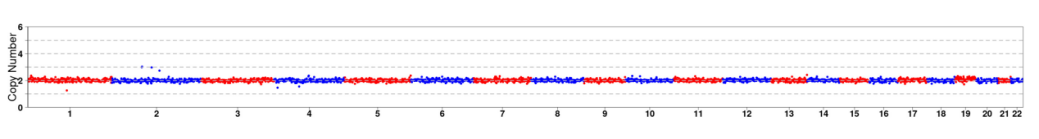 |  |
|  |  |  | BRD: Failed Detection |  |
|  |  |  | 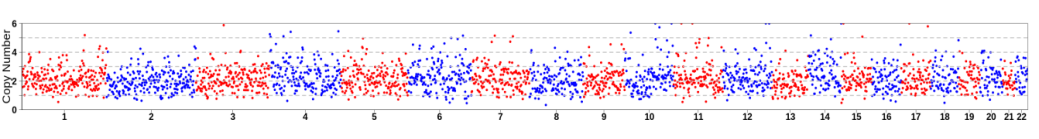 |  |
| B43 | D6 | 4CB | Biopsy Cells: +16 | 2 |
|  |  |  | 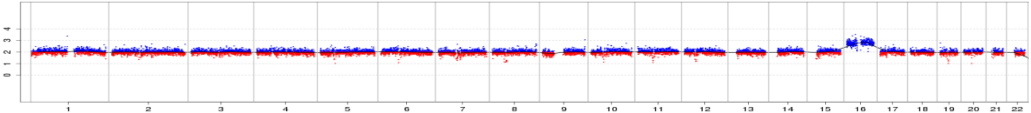 |  |
|  |  |  | BRD: +16 |  |
|  |  |  | 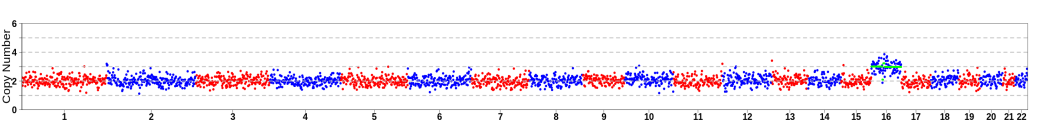 |  |
| B44 | D6 | 4CB | Biopsy Cells: -20 | 2 |
|  |  |  | 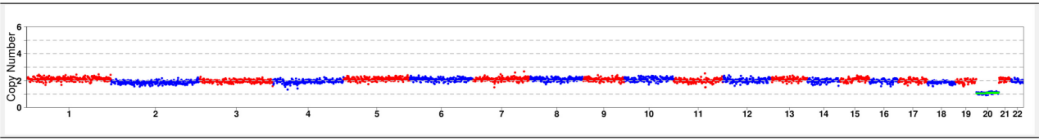 |  |
|  |  |  | BRD: -20(~56%) |  |
|  |  |  | 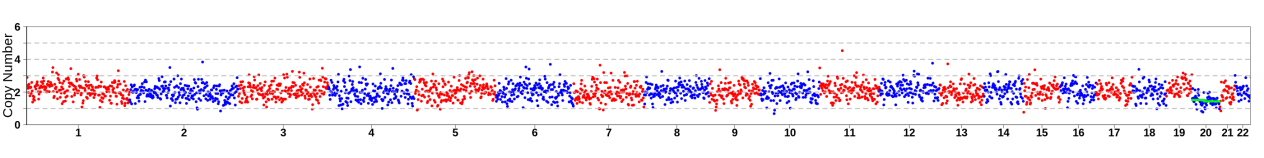 |  |
| B45 | D5 | 4BB | Biopsy Cells: Euploid | 2 |
|  |  |  | 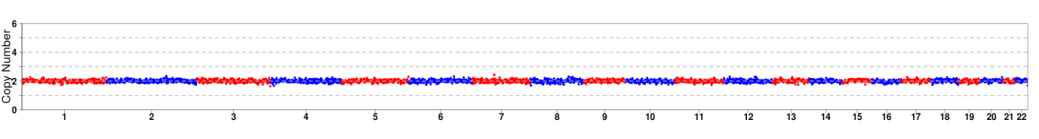 |  |
|  |  |  | BRD: Euploid |  |
|  |  |  | 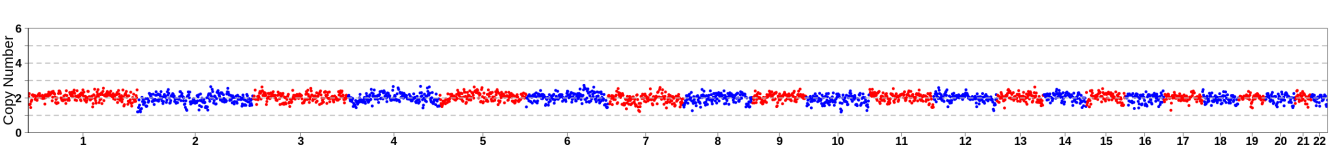 |  |
| B46 | D5 | 4BC | Biopsy Cells: Euploid | 2 |
|  |  |  | 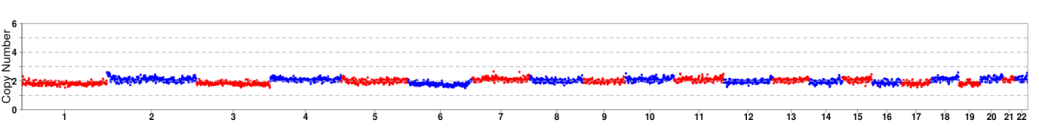 |  |
|  |  |  | BRD: Euploid |  |
|  |  |  | 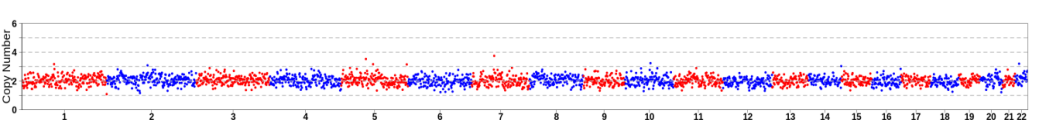 |  |
| B47 | D6 | 4BC | Biopsy Cells: +17,-21 | 2 |
|  |  |  | 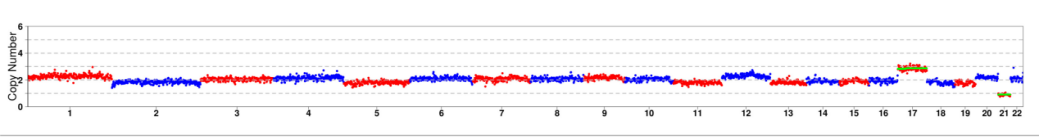 |  |
|  |  |  | BRD: +17(~38%),-21(~31%) |  |
|  |  |  | 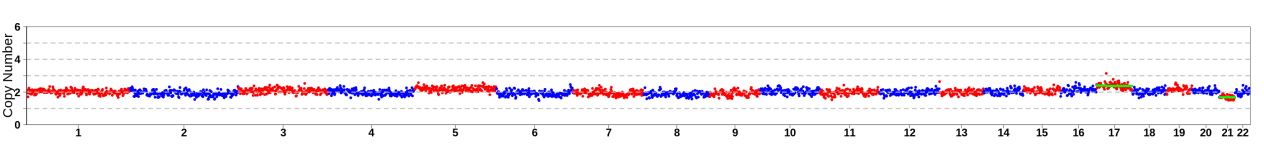 |  |
| B48 | D5 | 4BB | Biopsy Cells: Euploid | 2 |
|  |  |  | 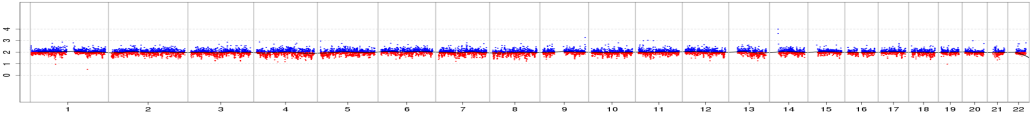 |  |
|  |  |  | BRD: Euploid |  |
|  |  |  | 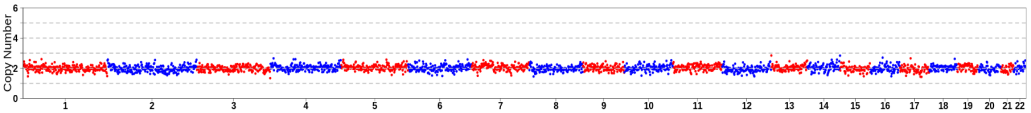 |  |
| B49 | D6 | 4BB | Biopsy Cells: Euploid | 2 |
|  |  |  | 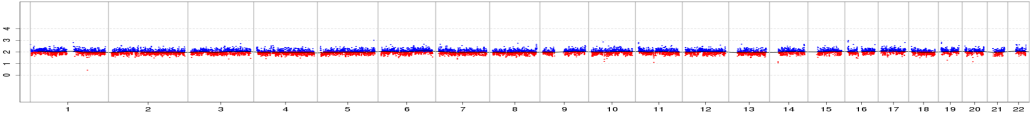 |  |
|  |  |  | BRD: Euploid |  |
|  |  |  | 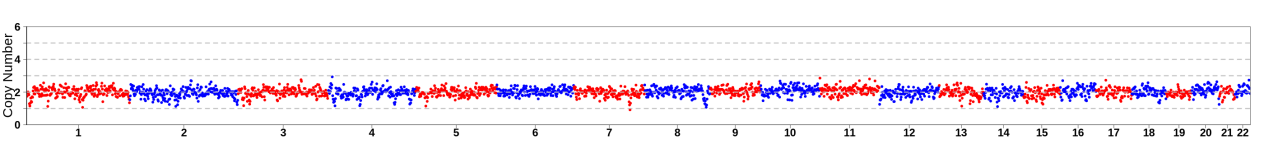 |  |
| B50 | D6 | 4BB | Biopsy Cells: +17,-20 | 2 |
|  |  |  | 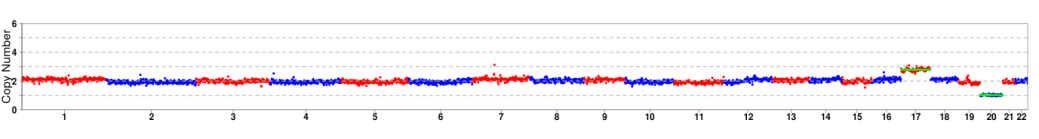 |  |
|  |  |  | BRD: +17(~53%),-20(~41%) |  |
|  |  |  | 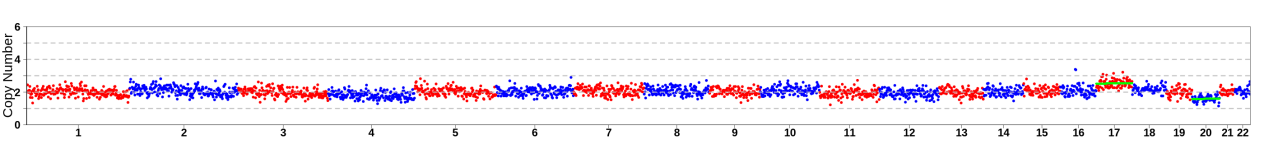 |  |
| B51 | D5 | 4BB | Biopsy Cells: Euploid | 2 |
|  |  |  | 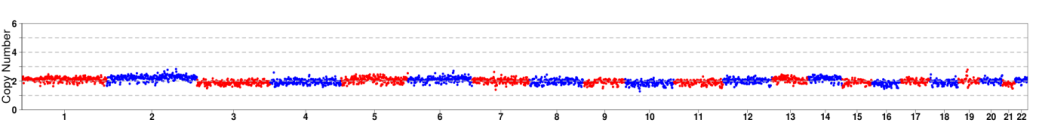 |  |
|  |  |  | BRD: Euploid |  |
|  |  |  | 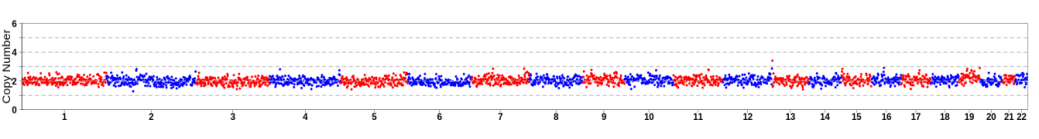 |  |
| B52 | D5 | 4BB | Biopsy Cells: Euploid | 2 |
|  |  |  | 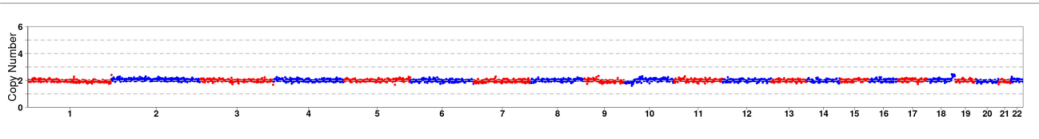 |  |
|  |  |  | BRD: Euploid |  |
|  |  |  | 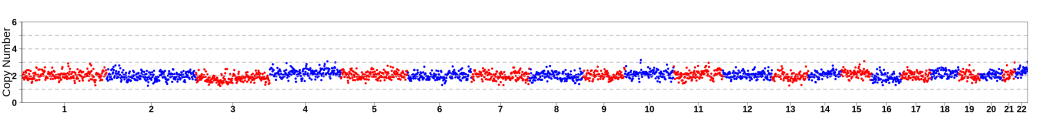 |  |
| B53 | D5 | 4BC | Biopsy Cells: Euploid | 2 |
|  |  |  | 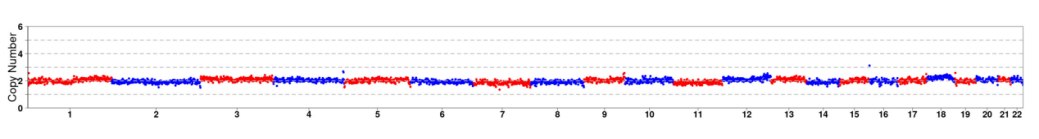 |  |
|  |  |  | BRD: Euploid |  |
|  |  |  | 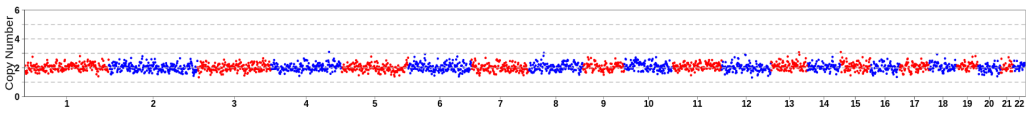 |  |
| B54 | D6 | 4AB | Biopsy Cells: Euploid | 2 |
|  |  |  | 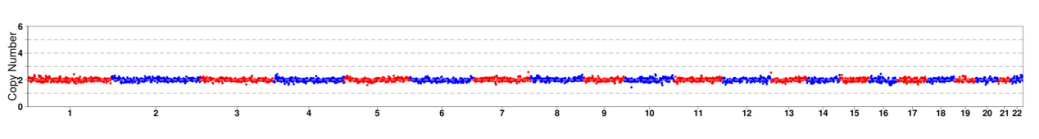 |  |
|  |  |  | BRD: Euploid |  |
|  |  |  | 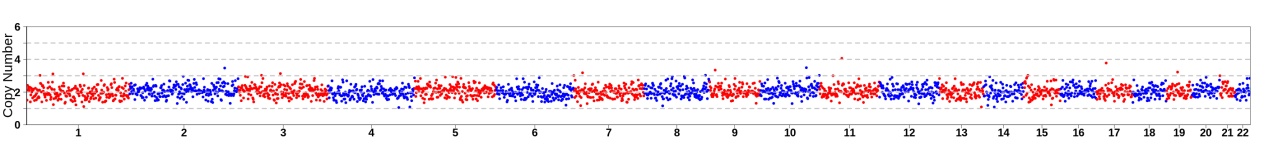 |  |
| B55 | D6 | 4BC | Biopsy Cells: Euploid | 2 |
|  |  |  | 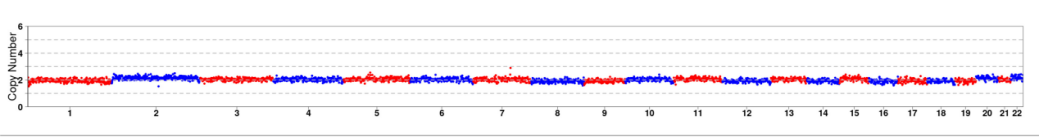 |  |
|  |  |  | BRD: Euploid |  |
|  |  |  | 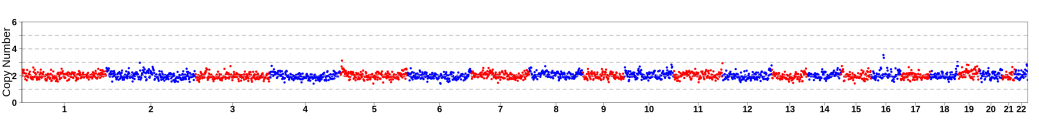 |  |
| B56 | D5 | 4BB | Biopsy Cells: Euploid | 2 |
|  |  |  | 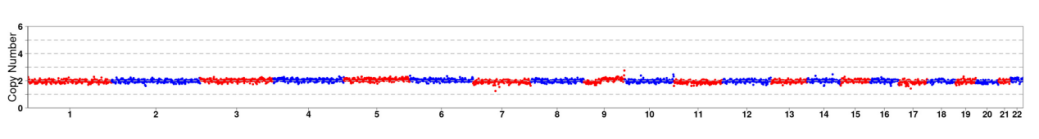 |  |
|  |  |  | BRD: Euploid |  |
|  |  |  | 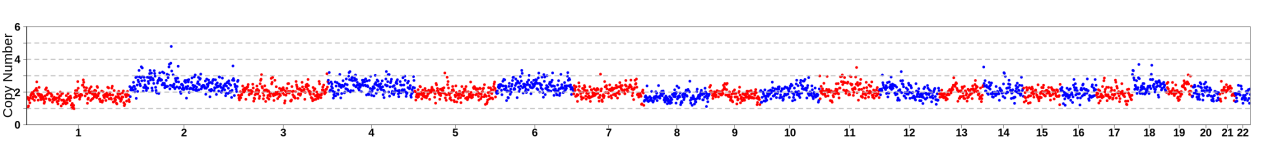 |  |
| B57 | D6 | 4B+B+ | Biopsy Cells: Euploid | 2 |
|  |  |  | 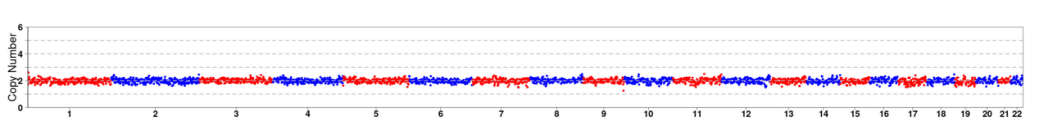 |  |
|  |  |  | BRD: Euploid |  |
|  |  |  | 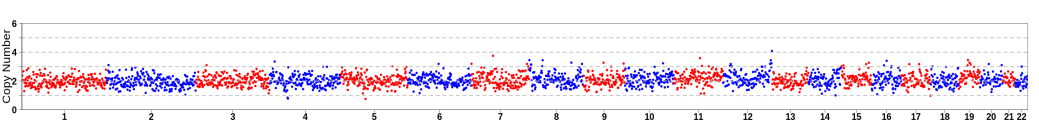 |  |
| B58 | D6 | 4AB | Biopsy Cells: +X(~32%),-Y(~33%) | 2 |
|  |  |  | 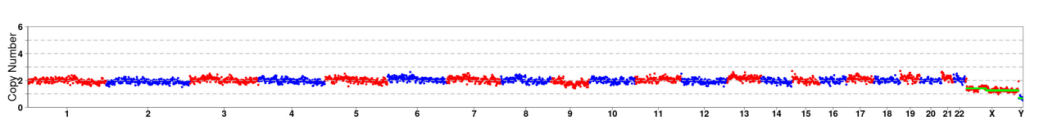 |  |
|  |  |  | BRD: +X(~40%),-Y(~37%) |  |
|  |  |  | 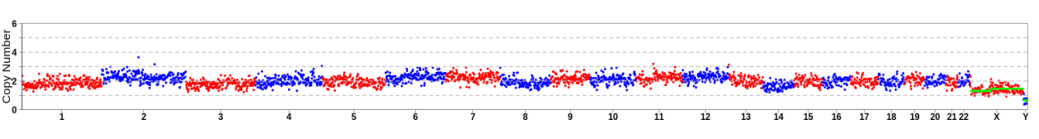 |  |
| B59 | D6 | 4BC | Biopsy Cells: Euploid | 2 |
|  |  |  | 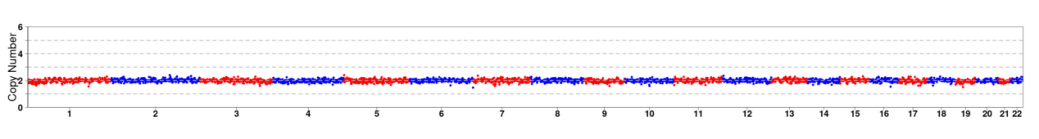 |  |
|  |  |  | BRD: Euploid |  |
|  |  |  | 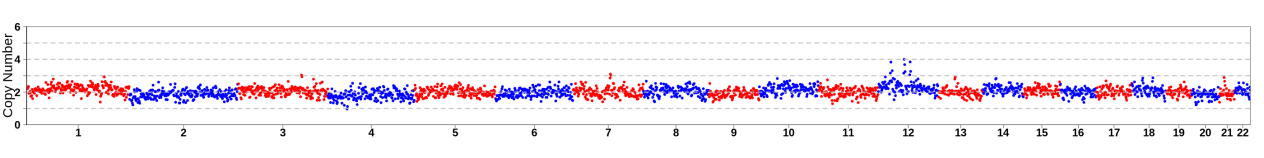 |  |
| B60 | D6 | 4BC | Biopsy Cells: -X,-14,+15 | 2 |
|  |  |  | 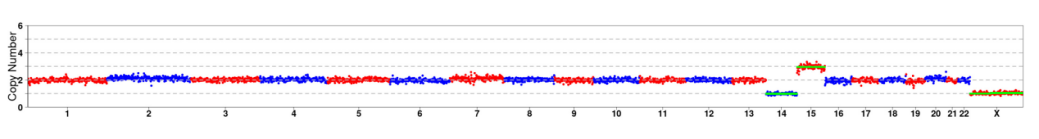 |  |
|  |  |  | BRD: -X(~50%),-14(~63%),+15 |  |
|  |  |  | 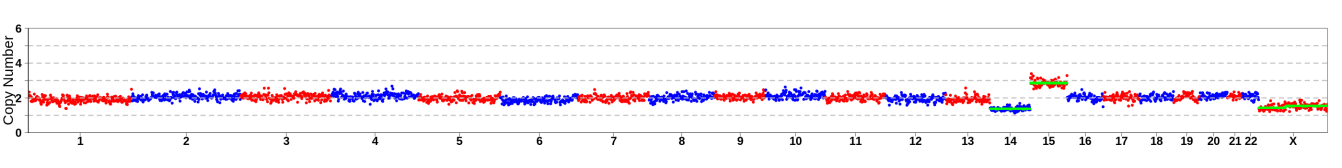 |  |
| B61 | D5 | 4BB | Biopsy Cells: Euploid | 2 |
|  |  |  | 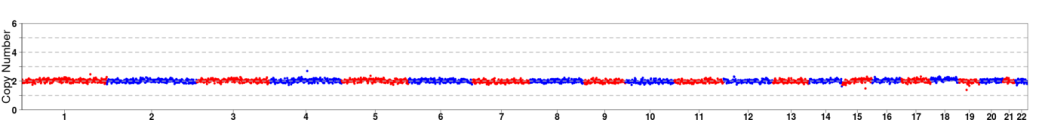 |  |
|  |  |  | BRD: Euploid |  |
|  |  |  | 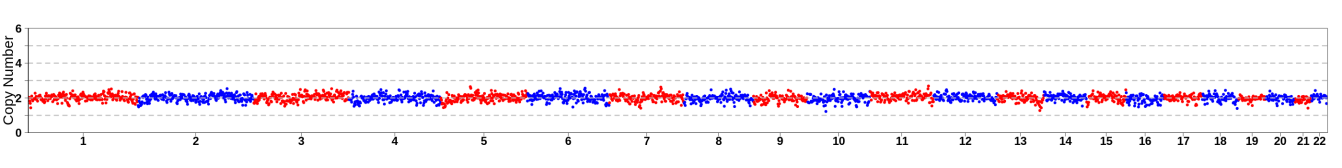 |  |
| B62 | D6 | 4BC | Biopsy Cells: -22 | 2 |
|  |  |  |  |  |
|  |  |  | BRD: -22 |  |
|  |  |  |  |  |
| B63 | D6 | 3BC | Biopsy Cells: Euploid | 2 |
|  |  |  |  |  |
|  |  |  | BRD: Euploid |  |
|  |  |  |  |  |
| B64 | D6 | 4BC | Biopsy Cells: Euploid | 2 |
|  |  |  |  |  |
|  |  |  | BRD: Euploid |  |
|  |  |  |  |  |
| B65 | D6 | 4BC | Biopsy Cells: Euploid | 2 |
|  |  |  |  |  |
|  |  |  | BRD: Euploid |  |
|  |  |  |  |  |
| B66 | D5 | 4BB | Biopsy Cells: del(7)(q32.3q36.3)(~26.74Mb,~25%) | 2 |
|  |  |  |  |  |
|  |  |  | BRD: del(7)(q32.3q36.3)(~27.64Mb) |  |
|  |  |  |  |  |
| B67 | D6 | 4BB | Biopsy Cells: +14,+18 | 2 |
|  |  |  |  |  |
|  |  |  | BRD: +14,+18 |  |
|  |  |  |  |  |
| B68 | D6 | 4BB | Biopsy Cells: Euploid | 2 |
|  |  |  |  |  |
|  |  |  | BRD: Euploid |  |
|  |  |  |  |  |
| B69 | D6 | 4BC | Biopsy Cells: +20,+21 | 2 |
|  |  |  |  |  |
|  |  |  | BRD: +20,+21 |  |
|  |  |  |  |  |
| B70 | D5 | 4BB | Biopsy Cells: Euploid | 2 |
|  |  |  |  |  |
|  |  |  | BRD: Euploid |  |
|  |  |  |  |  |
| B71 | D6 | 3BC | Biopsy Cells: -11 | 2 |
|  |  |  |  |  |
|  |  |  | BRD: -11(~43%) |  |
|  |  |  |  |  |
| B72 | D6 | 4BC | Biopsy Cells: +1(~55%),-2(~47%),+4(~52%),+5(~54%),+6(~55%),+8(~54%),+12(~55%),+17(~56%),+18(~64%),+22 | 1 |
|  |  |  |  |  |
|  |  |  | BRD: -X(~51%),+4(~69%),+6(~61%),-10(~63%),+12,-13(~48%),-15(~46%),+17(~48%),+18,-21(~43%),+22 |  |
|  |  |  |  |  |
| B73 | D6 | 4BC | Biopsy Cells: -3(~61%),+5(~43%),+10,-15,-18(~62%) | 1 |
|  |  |  |  |  |
|  |  |  | BRD: -9(~30%),-15,+22(~42%) |  |
|  |  |  |  |  |
| B74 | D6 | 3BC | Biopsy Cells: +2(~41%),-11 | 1 |
|  |  |  |  |  |
|  |  |  | BRD: dup(X)(q11.1q28)(~93.77Mb,~39%),dup(2)(p25.3p24.1)(~20.00Mb),dup(4)(q21.1q35.2)  (~112.65Mb),-5(~39%),dup(7)(q35q36.3)(~15.14Mb,~62%),+18(~40%) |  |
|  |  |  |  |  |
| B75 | D6 | 4BC | Biopsy Cells: del(3)(p26.3p21.1)(~53.60Mb,~51%),+5,+9,+12,+13,+15,+16,-18(~46%),+21,+22 | 1 |
|  |  |  |  |  |
|  |  |  | BRD: +16(~45%),+18(~49%) |  |
|  |  |  |  |  |
| B76 | D6 | 4BB | Biopsy Cells: +16 | 1 |
|  |  |  |  |  |
|  |  |  | BRD: -4,+16 |  |
|  |  |  |  |  |
| B77 | D6 | 4BB | Biopsy Cells: del(3)(p13-p11.1)(17Mb, 32%),-16(62%) | 1 |
|  |  |  |  |  |
|  |  |  | BRD: +X(~35%),-Y,dup(9)(q22.32q34.3)(~42.71Mb,~44%),dup(15)(q26.1q26.3)(~13.03Mb),-16(~61%),-21(~56%),dup(22)(q12.3q13.33)(~14.30Mb) |  |
|  |  |  |  |  |
| B78 | D6 | 4BC | Biopsy Cells: del(13)(q11q33.3)(~89.00Mb,~34%),del(13)(q33.3q34)(~5.57Mb),del(14)(q11.2q22.3)(~36.00Mb,~37%),del(14)(q22.3q32.33)(~51.35Mb),del(18)(q11.2q12.1)(~4.00Mb) | 1 |
|  |  |  |  |  |
|  |  |  | BRD: dup(13)(q11q31.3)(~73.50Mb),del(14)(q22.3q32.33)(~51.35Mb) |  |
|  |  |  |  |  |
| B79 | D6 | 4BB | Biopsy Cells: -14 | 1 |
|  |  |  |  |  |
|  |  |  | BRD: del(14)(q11.2q21.1)(~19.00Mb,~65%),dup(14)(q21.1q32.33)(~68.85Mb) |  |
|  |  |  |  |  |
| B80 | D6 | 4BC | Biopsy Cells: -8,-10,-21 | 1 |
|  |  |  |  |  |
|  |  |  | BRD: -8(~43%),-12(~40%),del(13)(q12.11q14.3)(~33.50Mb),dup(17)(q11.1q21.31)(~16.50Mb),-20(~49%) |  |
|  |  |  |  |  |
| B81 | D5 | 4BC | Biopsy Cells: -19 | 1 |
|  |  |  |  |  |
|  |  |  | BRD: +1(~59%),dup(6)(p25.3q21)(~111.50Mb,~46%),+10,-19,+22(~54%) |  |
|  |  |  |  |  |
| B82 | D6 | 4BC | Biopsy Cells: +6(~34%),-21 | 1 |
|  |  |  |  |  |
|  |  |  | BRD: +X(~54%),-Y(~48%),-21(~59%) |  |
|  |  |  |  |  |
| B83 | D5 | 4BC | Biopsy Cells: +10,+22 | 1 |
|  |  |  |  |  |
|  |  |  | BRD: +10,dup(18)(q22.1q23)(~11.58Mb),+22 |  |
|  |  |  |  |  |
| B84 | D5 | 4BB | Biopsy Cells: +22 | 1 |
|  |  |  |  |  |
|  |  |  | BRD: del(5)(p15.33p15.2)(~10.00Mb),del(8)(q12.1q23.3)(~56.00Mb),del(13)(q32.1q34)(~15.00Mb),del(19)(p13.3p13.2)(~11.50Mb),+22 |  |
|  |  |  |  |  |
| B85 | D6 | 4BC | Biopsy Cells: dup(12)(q21.1q21.32)(~13.00Mb,~54%),-16,dup(22)(q11.21q13.33)(~30.10Mb,~43%) | 1 |
|  |  |  |  |  |
|  |  |  | BRD: +3(~42%),-5(~45%),-16 |  |
|  |  |  |  |  |
| B86 | D6 | 4BC | Biopsy Cells: +2,+5(~63%),-11,+21,+22 | 1 |
|  |  |  |  |  |
|  |  |  | BRD: +2,-5,-11,+21,+22 |  |
|  |  |  |  |  |
| B87 | D7 | 4BC | Biopsy Cells: del(10)(p15.3p13)(~14.20Mb),-17(~38%),+18(~38%) | 0 |
|  |  |  |  |  |
|  |  |  | BRD: Euploid |  |
|  |  |  |  |  |
| B88 | D5 | 4AB | Biopsy Cells: +21 | 0 |
|  |  |  |  |  |
|  |  |  | BRD: Euploid |  |
|  |  |  |  |  |
| B89 | D5 | 4AB | Biopsy Cells: del(X)(p22.33p11.23)(~49.80Mb,~43%) | 0 |
|  |  |  |  |  |
|  |  |  | BRD: Euploid |  |
|  |  |  |  |  |
| B90 | D6 | 4CB | Biopsy Cells: +19 | 0 |
|  |  |  |  |  |
|  |  |  | BRD: Euploid |  |
|  |  |  |  |  |
| B91 | D6 | 4BB | Biopsy Cells: del(3)(q11.2q29)(~100.40Mb,~45%) | 0 |
|  |  |  |  |  |
|  |  |  | BRD: +X(~30%) |  |
|  |  |  |  |  |
| B92 | D6 | 4BC | Biopsy Cells: -X(~47%),-20(~47%) | 0 |
|  |  |  |  |  |
|  |  |  | BRD: del(5)(p15.1q35.3)(~162.92Mb) |  |
|  |  |  |  |  |
| B93 | D6 | 4BB | Biopsy Cells: +1(~35%),+4(~34%),+6(~30%),-8,+22(~37%) | 0 |
|  |  |  |  |  |
|  |  |  | BRD: -2(~44%),-6(~48%),+8 |  |
|  |  |  |  |  |
| B94 | D6 | 4BC | Biopsy Cells: dup(10)(p15.3p11.21)(~37.20Mb,~38%),del(12)(p13.33q23.3)(~105.60Mb,~34%) | 0 |
|  |  |  |  |  |
|  |  |  | BRD: +X(~56%),-Y,dup(3)(p26.3p24.3)(~19.50Mb),dup(11)(q13.2q22.1)(~30.00Mb),dup(12)(p13.31p11.22)(~21.50Mb),dup(18)(q11.1q12.3)(~24.00Mb),dup(18)(q22.1q23)(~12.58Mb),+21 |  |
|  |  |  |  |  |
| B95 | D6 | 4BC | Biopsy Cells: +11,+13,-15,-21 | 0 |
|  |  |  |  |  |
|  |  |  | BRD: +X(~45%),-Y(~53%) |  |
|  |  |  |  |  |
| B96 | D6 | 4BC | Biopsy Cells: Euploid | 0 |
|  |  |  |  |  |
|  |  |  | BRD: +X(~69%),-Y,-3(~42%),-6(~42%) |  |
|  |  |  |  |  |
| B97 | D6 | 4BB | Biopsy Cells: dup(4)(p16.3q21.1)(~77.00Mb,~39%),del(4)(q21.1q35.2)(~114.20Mb,~66%) | 0 |
|  |  |  |  |  |
|  |  |  | BRD: +X,-Y |  |
|  |  |  |  |  |
| B98 | D6 | 4BB | Biopsy Cells: del(Y)(q11.21q11.223)(~9.80Mb) | 0 |
|  |  |  |  |  |
|  |  |  | BRD: -11 |  |
|  |  |  |  |  |
| B99 | D5 | 4BC | Biopsy Cells: del(12)(p13.33q24.32)(~126.60Mb,~51%),del(12)(q24.32q24.33)(~7.45Mb) | 0 |
|  |  |  |  |  |
|  |  |  | BRD: dup(X)(q11.1q21.31)(~30.00Mb,~48%),+20(~45%),+21(~68%) |  |
|  |  |  |  |  |
| B100 | D6 | 4BB | Biopsy Cells: del(4)(p16.3q12)(~53.00Mb,~36%),del(8)(q22.2q24.3)(~45.96Mb,~33%) | 0 |
|  |  |  |  |  |
|  |  |  | BRD: Euploid |  |
|  |  |  |  |  |
| B101 | D6 | 4AA | Biopsy Cells: +21 | 0 |
|  |  |  |  |  |
|  |  |  | BRD: +X,-Y |  |
|  |  |  |  |  |
| B102 | D6 | 4BB | Biopsy Cells: Euploid | 0 |
|  |  |  |  |  |
|  |  |  | BRD: del(9)(q21.11q34.3)(~70.71Mb,~46%),del(11)(q12.3q25)(~72.01Mb,~32%),del(16)(p13.13q24.3)(~79.35Mb,~43%),del(17)(q22q25.3)(~25.20Mb,~35%) |  |
|  |  |  |  |  |
| B103 | D5 | 4BB | Biopsy Cells: -X(~30%),-15(~34%) | 0 |
|  |  |  |  |  |
|  |  |  | BRD: +X(~45%),-Y,-6(~30%) |  |
|  |  |  |  |  |
| B104 | D6 | 5BB | Biopsy Cells: dup(10)(q24.32q26.3)(~32.33Mb,~62%) | 0 |
|  |  |  |  |  |
|  |  |  | BRD: dup(16)(q11.2q24.3)(~44.35Mb,~35%),-18(~50%) |  |
|  |  |  |  |  |
| B105 | D6 | 4BB | Biopsy Cells: -X | 0 |
|  |  |  |  |  |
|  |  |  | BRD: +X(~57%),+10(~41%),+15(~43%),+16(~43%),+18(~41%),-20 |  |
|  |  |  |  |  |
| B106 | D6 | 4BB | Biopsy Cells: Euploid | 0 |
|  |  |  |  |  |
|  |  |  | BRD: dup(X)(p22.33q25)(~120.00Mb,~67%),-1(~67%),+6(~46%),+8(~45%),+11(~43%),+13(~42%),+15(~47%),+16(~40%),+17(~40%),+18(~44%),-20(~59%),+21(~53%),+22(~43%) |  |
|  |  |  |  |  |
| B107 | D5 | 4BB | Biopsy Cells: Euploid | — |
|  |  |  |  |  |
|  |  |  | BRD: Detection Failed |  |
|  |  |  |  |  |

Note: CNV: Copy number variation; BRD: Biopsy-related droplets. “—” indicates data not applicable.

**Supplementary Table 2** BRD Detection Results in Euploid and Mosaic Blastocysts identified by PGT-A

| BRD Result Category | | Euploid Blastocysts (n = 72) | | Mosaic Blastocysts (n = 43) | |
| --- | --- | --- | --- | --- | --- |
|  |  | Count | Proportion (%) | Count | Proportion (%) |
| LCSL | | 46 | 63.89 | 22 | 51.16 |
| HCSL | Detection Failed | 4 | 5.56 | 3 | 6.98 |
|  | Euploid | 13 | 18.06 | 5 | 11.63 |
|  | Non-euploid | 9 | 12.50 | 13 | 30.23 |

Note: BRD: Biopsy-related droplets; CSL: Concentration of the sequencing library; PGT-A: Preimplantation genetic testing for aneuploidy. LCSL indicates CSL < 0.5 ng/μL; HCSL indicates CSL ≥ 0.5 ng/μL.
